# Predicting distant metastasis and chemotherapy benefit in locally advanced rectal cancer

**DOI:** 10.1038/s41467-020-18162-9

**Published:** 2020-08-27

**Authors:** Zhenyu Liu, Xiaochun Meng, Hongmei Zhang, Zhenhui Li, Jiangang Liu, Kai Sun, Yankai Meng, Weixing Dai, Peiyi Xie, Yingying Ding, Meiyun Wang, Guoxiang Cai, Jie Tian

**Affiliations:** 1 CAS Key Laboratory of Molecular Imaging, Beijing Key Laboratory of Molecular Imaging, The State Key Laboratory of Management and Control for Complex Systems, Institute of Automation, Chinese Academy of Sciences, 100190 Beijing, China; 2 CAS Center for Excellence in Brain Science and Intelligence Technology, Institute of Automation, Chinese Academy of Sciences, 100190 Beijing, China; 3 School of Artificial Intelligence, University of Chinese Academy of Sciences, 100080 Beijing, China; 4 Department of Radiology, The Sixth Affiliated Hospital of Sun Yat-sen University, Guangzhou, 510655 China; 5 Department of Diagnostic Radiology, National Cancer Center/National Clinical Research Center for Cancer/Cancer Hospital, Chinese Academy of Medical Sciences and Peking Union Medical College, 100021 Beijing, China; 6 Department of Radiology, The Third Affiliated Hospital of Kunming Medical University (Yunnan Cancer Hospital), Kunming, 650031 China; 7 Beijing Advanced Innovation Center for Big Data-Based Precision Medicine, School of Medicine and Engineering, Beihang University, 100191 Beijing, China; 8 Engineering Research Center of Molecular and Neuro Imaging of Ministry of Education, School of Life Science and Technology, Xidian University, Xi’an, 710126 China; 9 Department of Colorectal Surgery, Fudan University Shanghai Cancer Center, Shanghai, 200032 China; 10 Department of Radiology, Henan Provincial People’s Hospital & the People’s Hospital of Zhengzhou University, Zhengzhou, 450003 China

**Keywords:** Cancer imaging, Colorectal cancer

## Abstract

Distant metastasis (DM) is the main cause of treatment failure in locally advanced rectal cancer. Adjuvant chemotherapy is usually used for distant control. However, not all patients can benefit from adjuvant chemotherapy, and particularly, some patients may even get worse outcomes after the treatment. We develop and validate an MRI-based radiomic signature (RS) for prediction of DM within a multicenter dataset. The RS is proved to be an independent prognostic factor as it not only demonstrates good accuracy for discriminating patients into high and low risk of DM in all the four cohorts, but also outperforms clinical models. Within the stratified analysis, good chemotherapy efficacy is observed for patients with pN2 disease and low RS, whereas poor chemotherapy efficacy is detected in patients with pT1–2 or pN0 disease and high RS. The RS may help individualized treatment planning to select patients who may benefit from adjuvant chemotherapy for distant control.

## Abstract (web summary)

Distant metastasis (DM) is the main cause of treatment failure in locally advanced rectal cancer. Here, the authors developed and validated a radiomic signature (RS) for prediction of DM within a multicenter dataset, and suggest that it may help with stratification of patients who might benefit from adjuvant chemotherapy for DM.

## Introduction

Locally advanced rectal cancer (LARC) is the most common form of rectal cancer. Although a combination of neoadjuvant chemoradiotherapy and total mesorectal excision (TME) decrease locoregional recurrence rate to <5–10%, it has not noticeably increased survival^1–3^. Distant metastasis (DM) is the main cause of treatment failure in patients with LARC, as the incidence of DM remained 25–40%^4,5^. To reduce the incidence of DM, the guidelines of rectal cancer recommended adjuvant chemotherapy following TME for patients with LARC. However, adjuvant chemotherapy may only reduce the risk of DM and provide additional survival benefit in certain subsets of patients. Therefore, it is crucial how to detect the LARC patients that could benefit from adjuvant chemotherapy.

In fact, it is controversial which patients could benefit from adjuvant chemotherapy. The European Society for Medical Oncology clinical practice guidelines^6^ suggest that adjuvant therapy is unnecessary in pN0 cases if the patients does not receive neoadjuvant therapy. A pooled analysis has also indicated that pathological complete response (pCR, T0N0M0) patients would not benefit from adjuvant chemotherapy^7^. However, a recent study revealed conflicting evidence regarding whether adjuvant chemotherapy could improve overall survival in patients with pCR^8^. While it is difficult to stratify patients based on the traditional TNM staging system, Valentini’s nomogram (VN) has been developed based on clinical prognostic factors to identify patients who may benefit from adjuvant chemotherapy^9^. Although this model fulfilled the predefined criteria for American Joint Committee on Cancer endorsement^10^, it ignores the potentially pathological risk factors like lymphovascular invasion (LVI) and perineural invasion (PNI) as well as more comprehensive information that can be obtained from multiparametric MRI. Incorporating these factors, the prediction model might achieve better performance for detecting patients at high risk of DM.

Nowadays, MRI is widely used for diagnosing and staging of rectal cancer, and can detect several prognostic factors^11,12^. Furthermore, radiomic analysis of these images may provide prognostic information, as medical images can provide not only structural information but also information regarding the underlying pathophysiology, which may be associated with the patient’s prognosis^13–15^. Thus, radiomics has been successfully used to improve diagnostic accuracy^16^, evaluate response to neoadjuvant therapy^17,18^, and predict prognosis^19^. Moreover, radiomics may, in theory, help relate the patient’s prognosis to quantitative imaging features that objectively describe the tumor’s nature^20^. Specifically, MRI-based radiomics has been proved to be an effective tool for the prediction of preoperative synchronous DM in patients with rectal cancer^21^. However, there were still few studies focusing on the prediction of postoperative DM after surgery and adjuvant chemotherapy benefit.

In the present study, we investigate the imaging features associated with the prognosis of LARC patients, and then develop and validate a model to predict DM after surgery. With this model, we further identify patients who can benefit from adjuvant chemotherapy.

## Results

### Patient characteristics

The characteristics of the 629 enrolled patients are shown in Table 1. The median follow-up time for distant metastasis-free survival (DMFS) were 49.6 months in the primary cohort (interquartile range [IQR]: 47.3–52.6 months), 52.6 months in validation cohort 1 (IQR: 50.3–58.9 months), 43.1 months in validation cohort 2 (IQR: 42.3–45.7 months), and 46.3 months in validation cohort 3 (IQR: 45.2–48.2 months).

**Table 1:** Demographic and clinicopathological characteristics.

		Primary cohort (*n* = 176)	Validation cohort 1 (*n* = 154)	Validation cohort 2 (*n* = 150)	Validation cohort 3 (*n* = 149)
Age (years, mean ± SD)		57.3 ± 12.7	55.7 ± 12.4	56.3 ± 10.1	56.6 ± 11.7
Sex (%)	Male	108 (61.4%)	101 (65.6%)	96 (64.0%)	101 (67.8%)
	Female	68 (38.6%)	53 (34.4%)	54 (36.0%)	48 (32.2%)
Clinical stage (%)	II	87 (49.4%)	23 (14.9%)	42 (28.0%)	53 (35.6%)
	III	89 (50.6%)	131 (85.1%)	108 (72.0%)	96 (64.4%)
Clinical T stage (%)	2	9 (5.1%)	2 (1.3%)	0	0
	3	91 (51.7%)	108 (70.1%)	116 (77.3%)	119 (79.9%)
	4	76 (43.2%)	44 (28.6%)	34 (22.7%)	30 (20.1%)
Lymph node status (%)	LN negative	84 (47.7%)	25 (16.2%)	42 (28.0%)	37 (24.8%)
	LN positive	92 (52.3%)	129 (83.8%)	108 (72.0%)	112 (75.2%)
Location (%)	>10 cm	31 (17.6%)	103 (66.9%)	11 (7.3%)	12 (8.1%)
	5–10 cm	65 (36.9%)	50 (32.5%)	81 (54.0%)	93 (62.4%)
	<5 cm	80 (45.5%)	1 (0.6%)	58 (38.7%)	44 (29.5%)
Preoperative serum CEA (%)	<5	109 (61.9%)	81 (52.6%)	114 (76.0%)	98 (65.8%)
	≥5	67 (38.1%)	73 (47.4%)	36 (24.0%)	51 (34.2%)
Neoadjuvant therapy (%)	Yes	63 (35.8%)	154 (100%)	81 (54.0%)	86 (57.7%)
	No	113 (64.2%)	0	69 (46.0%)	63 (42.3%)
Adjuvant chemotherapy (%)	Yes	117 (66.5%)	51 (33.1%)	123 (82.0%)	115 (77.2%)
	No	59 (33.5%)	103 (66.9%)	27 (18.0%)	34 (22.8%)
Adjuvant radiotherapy (%)	Yes	10 (5.7%)	1 (0.6%)	26 (17.3%)	3 (2.0%)
	No	166 (94.3%)	153 (99.4%)	124 (82.7%)	146 (98.0%)
Surgery procedure (%)	Dixon + preventive ileostomy	27 (15.3%)	26 (16.9%)	24 (16.0%)	38 (25.5%)
	Dixon	83 (47.2%)	45 (29.2%)	63 (42.0%)	97 (65.1%)
	Miles	53 (30.1%)	77 (50.0%)	53 (35.3%)	13 (8.7%)
	Hartmann	13 (7.4%)	6 (3.9%)	10 (6.7%)	1 (0.7%)
Surgery approach (%)	Open resection	119 (67.6%)	52 (33.8%)	129 (86.0%)	9 (6.0%)
	Laparoscopic resection	57 (32.4%)	102 (66.2%)	21 (14.0%)	140 (94.0%)
pN stage (%)	0	80 (45.5%)	92 (59.7%)	86 (57.3%)	101 (67.8%)
	1	66 (37.5%)	43 (27.9%)	41 (27.3%)	24 (16.1%)
	2	30 (17.0%)	19 (12.3%)	23 (15.3%)	24 (16.1%)
pT stage (%)	0	4 (2.3%)	35 (22.7%)	27 (18.0%)	0
	1/2	24 (13.6%)	30 (19.5%)	26 (17.3%)	26 (17.4%)
	3/4	148 (84.1%)	89 (57.8%)	97 (64.7%)	123 (82.6%)
LVI (%)	Positive	51 (29.0%)	6 (3.9%)	25 (16.7%)	12 (8.1%)
	Negative	125 (71.0%)	148 (96.1%)	125 (83.3%)	137 (91.9%)
PNI (%)	Positive	18 (10.2%)	15 (9.7%)	27 (18%)	11 (7.4%)
	Negative	158 (89.8%)	139 (90.3%)	123 (82%)	138 (92.6%)

*CEA* carcinoembryonic antigen, *pN* pathological nodal stage, *pT* pathological tumor stage, *LVI* lymphovascular invasion, *PNI* perineural invasion.

Satisfactory inter- and intra-observer reproducibility were observed for the tumor masking and radiomic feature extraction (ICC > 0.6)^22^ when we compared results for five radiologists and results from the same radiologist at baseline and at least 1 month later.

### Radiomic signature construction and validation

**Fig. 1:**
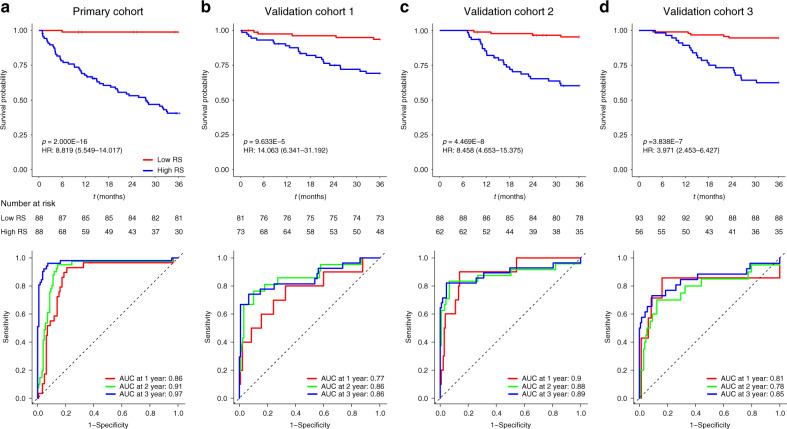
K–M and time-dependent ROC curves according to the RS. *p* values were calculated using two-sided log-rank test, and AUCs at 1 year, 2 years, and 3 years were calculated to assess the prognostic accuracy within the primary cohort (**a**: *n* = 176), validation cohort 1 (**b**: *n* = 154), validation cohort 2 (**c**: *n* = 150), and validation cohort 3 (**d**: *n* = 149). AUC area under the curve; HR hazard ratio; ROC receiver operating characteristic; RS radiomic signature. Source data are provided as a Source Data file.

**Fig. 2:**
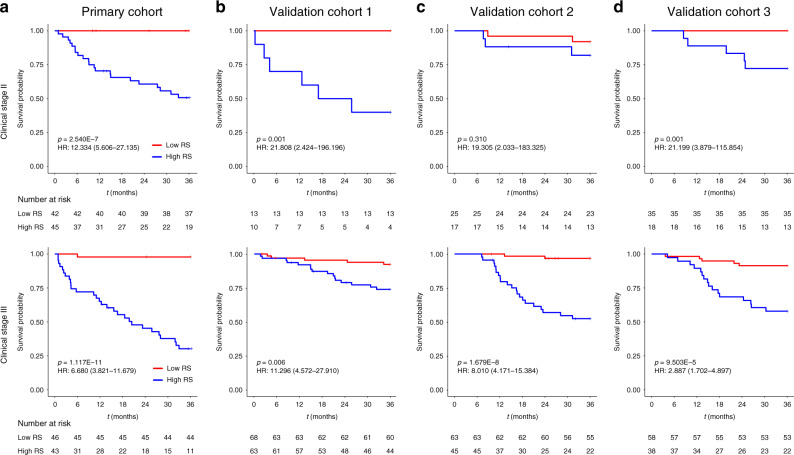
K–M DMFS curves according to the RS among LARC patient subgroups. **a** The primary cohort (upper: stage II, *n* = 87; lower: stage III, *n* = 89). **b** Validation cohort 1 (upper: stage II, *n* = 23; lower: stage III, *n* = 131). **c** Validation cohort 2 (upper: stage II, *n* = 42; lower: stage III, *n* = 108). **d** Validation cohort 3 (upper: stage II, *n* = 53; lower: stage III, *n* = 96). *p* values were calculated using two-sided log-rank test. RS radiomic signature; HR hazard ratio; DMFS distant metastasis-free survival; LARC locally advanced rectal cancer. Source data are provided as a Source Data file.

The coarse-to-fine feature selection strategy identified four relevant features (Supplementary Table 1). The selected features were incorporated into a least absolute shrinkage and selection operator (LASSO)-Cox regression model to define the radiomic signature. For each of the primary cohort and the three validation cohorts, patients were classified into high- and low-radiomic signature groups for further analyses based on the median radiomic signature value of the primary cohort. The Kaplan–Meier survival curves confirmed a significant difference in DMFS between the high- and low-radiomic signature groups (*p* < 0.001), with relatively high hazard ratios (HRs, >3.9) in all four cohorts (Fig. 1a–d, upper). The areas under the curve (AUCs) at different follow-up times (1, 2, and 3 years) also confirmed that the radiomic signature had good prognostic accuracy in the primary and validation cohorts (Fig. 1a–d, lower). Subgroup analyses further confirmed that the radiomic signature could predict prognosis according to clinical stage (Fig. 2) as well as in the pT and pN subgroups from each cohort (Supplementary Figs. 1 and 2). These results confirmed the high prognostic accuracy of the radiomic signature.

### Incremental value of the radiomic signature

**Fig. 3:**
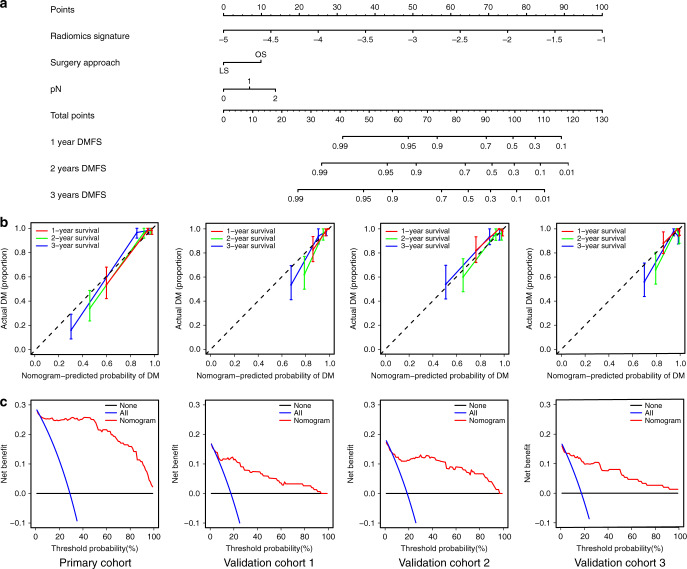
Nomogram, calibration curves, and decision curves to estimate DMFS. **a** The radiomic nomogram for estimating DMFS. **b** The calibration curves for the radiomic nomogram in the primary and validation cohorts (left to right: the primary cohort with *n* = 176, validation cohort 1 with *n* = 154, validation cohort 2 with *n* = 150, and validation cohort 3 with *n* = 149). The error bars were defined as s.e.m., which represent the 95% CI. **c** The decision curves for the nomogram in the primary and validation cohorts (left to right: the primary cohort with *n* = 176, validation cohort 1 with *n* = 154, validation cohort 2 with *n* = 150, and validation cohort 3 with *n* = 149). DMFS distant metastasis-free survival.

Multivariate Cox analysis revealed that DM was independently predicted by the radiomic signature, surgery location, and pN stage. Therefore, a radiomic nomogram (Fig. 3a) and clinical models (Supplementary Fig. 3) were constructed using the primary cohort. The calibration curves for the radiomic nomogram at 1 year, 2 years, and 3 years showed good agreement between the estimations and the clinical outcomes in the primary and validation cohorts. The C-index values for the different models, namely radiomic signature, radiomic nomogram, clinical nomogram, and VN, are listed in Table 2. Relative to the clinical nomogram and the VN, the radiomic signature provided better performance in the primary cohort (C-index: 0.847, 95% confidence interval [CI]: 0.803–0.891) and the validation cohorts (validation cohort 1: C-index: 0.809, 95% CI: 0.718–0.901; validation cohort 2: C-index: 0.848, 95% CI: 0.761–0.934; validation cohort 3: C-index: 0.803, 95% CI: 0.705–0.901) (Table 2). Furthermore, the radiomic nomogram based on the radiomic signature and clinicopathologic factors (Supplementary Table 2) also achieved better performance and significantly improved the classification accuracy for DMFS outcomes, based on the net reclassification improvement (NRI) and integrated discrimination improvement (IDI) values (Supplementary Fig. 4).

**Table 2:** The performances of the different models in the primary and validation cohorts.

Cohort	C-index (95% CI)			
	Radiomic signature	Clinical nomogram	Radiomic nomogram	Valentini’s nomogram
Primary Cohort	0.847 (0.803–0.891)	0.682 (0.618–0.745)	0.855 (0.812–0.899)	0.686 (0.620–0.751)
Validation Cohort 1	0.809 (0.718–0.901)	0.595 (0.483–0.706)	0.848 (0.773–0.923)	0.707 (0.628–0.786)
Validation Cohort 2	0.848 (0.761–0.934)	0.508 (0.405–0.612)	0.831 (0.742–0.920)	0.495 (0.391–0.598)
Validation Cohort 3	0.803 (0.705–0.901)	0.631 (0.532−0.730)	0.825 (0.728−0.921)	0.644 (0.537−0.751)

*C-index* concordance index, *CI* confidence interval.

The decision curve analysis revealed that the radiomic nomogram had relatively good clinical performance, with advantages across almost the entire range of reasonable threshold probabilities in the primary and validation cohorts.

These results suggested that the radiomic signature provided additional value for personalized DM prediction.

### Risk stratification using the radiomic signature

In order to detect patients that can benefit from adjuvant chemotherapy, interaction tests among radiomic signature, pathological stage, and adjuvant chemotherapy efficacy were performed (Table 3).

**Table 3:** Treatment interaction with radiomic signature and pathological stage for DMFS in patients with LARC.

	CT	No CT	Distant metastases-free survival		
			CT vs NO CT, HR (95%CI)	*p*	*p* value for interaction
All (*n* = 629)					
High RS	176	103	1.71 (1.13–2.57)	0.01	2.54 × 10^−37^
Low RS	230	120	0.75 (0.27–2.12)	0.59	
pT = 0 (*n* = 66)					
High RS	13	10	4.22 (0.49–36.16)	0.15	0.102
Low RS	24	19	1.55 (0.14–17.09)	0.72	
pT = 1/2 (*n* = 106)					
High RS	29	20	11.66 (1.53–88.83)	0.003	4.13 × 10^−13^
Low RS	33	24	NA	0.23	
pT = 3/4 (*n* = 457)					
High RS	134	73	1.25 (0.81–1.93)	0.32	5.09 × 10^−29^
Low RS	173	77	0.76 (0.22–2.59)	0.66	
pN = 0 (*n* = 359)					
High RS	83	58	2.67 (1.27–5.60)	0.007	1.43 × 10^−11^
Low RS	130	88	1.33 (0.24–7.26)	0.74	
pN = 1 (*n* = 174)					
High RS	58	27	1.15 (0.57–2.30)	0.70	2.76 × 10^−17^
Low RS	68	21	0.91 (0.09–8.70)	0.93	
pN = 2 (*n* = 96)					
High RS	35	18	1.16 (0.57–2.35)	0.68	1.67 × 10^−31^
Low RS	32	11	0.18 (0.03–1.06)	0.03	

*RS* radiomic signature, *CT* chemotherapy, *HR* hazard ratio, *CI* confidence interval.

*p* values were calculated using two-sided log-rank test.

**Fig. 4:**
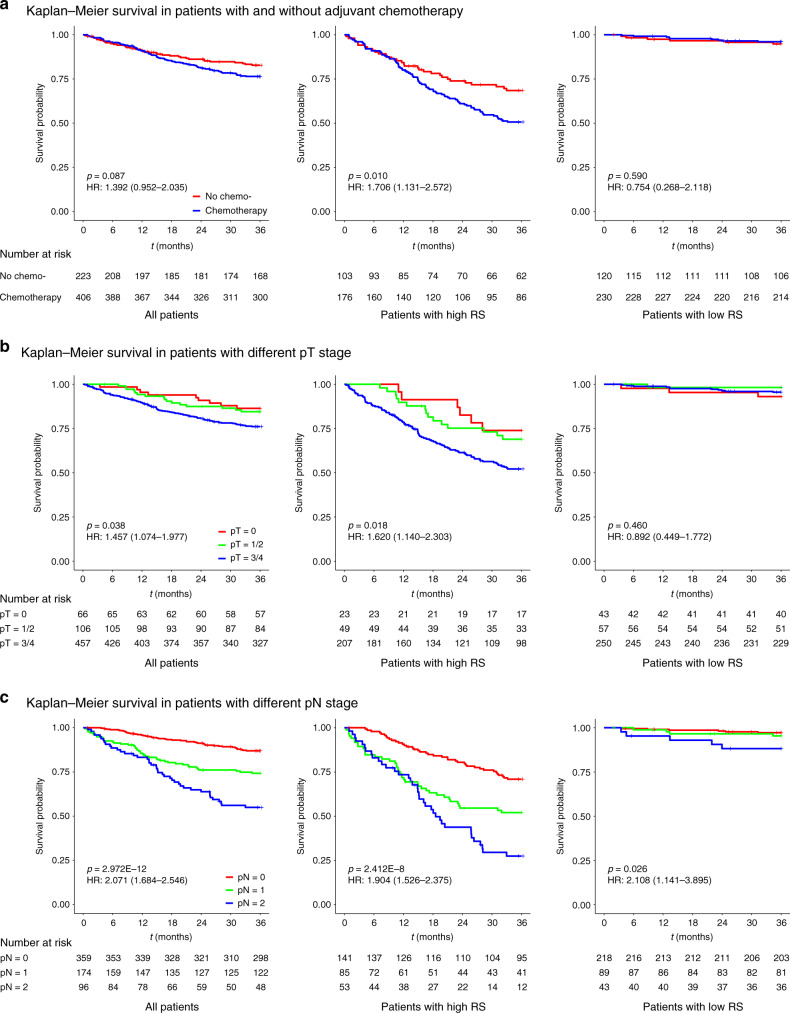
K–M DMFS curves for patients with LARC according to the RS. The results are shown for all patients (*n* = 629, left), patients with a high RS (*n* = 279, middle), and patients with a low RS (*n* = 350, right). The results are also stratified according to adjuvant chemotherapy use (**a**), pT stage (**b**), and pN stage (**c**). *p* values were calculated using two-sided log-rank test. RS radiomic signature; HR hazard ratio; DMFS distant metastasis-free survival; LARC locally advanced rectal cancer. Source data are provided as a Source Data file.

The interaction test for radiomic signature and adjuvant chemotherapy efficacy revealed that the adjuvant chemotherapy benefit was worse among patients with a high-radiomic signature (HR: 1.706, 95% CI: 1.131–2.572, *p* < 0.05; *p* < 0.001 for interaction), relative to among patients with a low-radiomic signature. The corresponding Kaplan–Meier DMFS curves are shown for the high- and low-radiomic signature groups in Fig. 4a. Adjuvant chemotherapy was significantly associated with decreased DMFS in the high-radiomic signature group (*p* = 0.01), did not have a significant association in the low-radiomic signature group, and had only a marginally significant association among all patients (*p* = 0.087) (Fig. 4a). These results suggest that LARC patients with a high-radiomic signature may experience even worse outcomes after receiving adjuvant chemotherapy.

The interaction tests for radiomic signature and pathological stage revealed that both pT stage and pN stage were associated with DMFS among all patients (Fig. 4b, c). Advanced stages suggested high risk of DM, meaning that patients with higher stage usually showed decreased DMFS. Specifically, pT stage was significantly associated with DMFS in the high-radiomic signature group (HR: 1.620, 95% CI: 1.140–2.303, *p* < 0.05) not in the low-radiomic signature group, and pN stage was significantly associated with DMFS in both the high- and low-radiomic signature groups (HR: 1.904, 95% CI: 1.526–2.375, *p* < 0.05 in high-radiomic signature group, and HR: 2.108, 95% CI: 1.141–3.895, *p* < 0.05 in low-radiomic signature group).

**Fig. 5:**
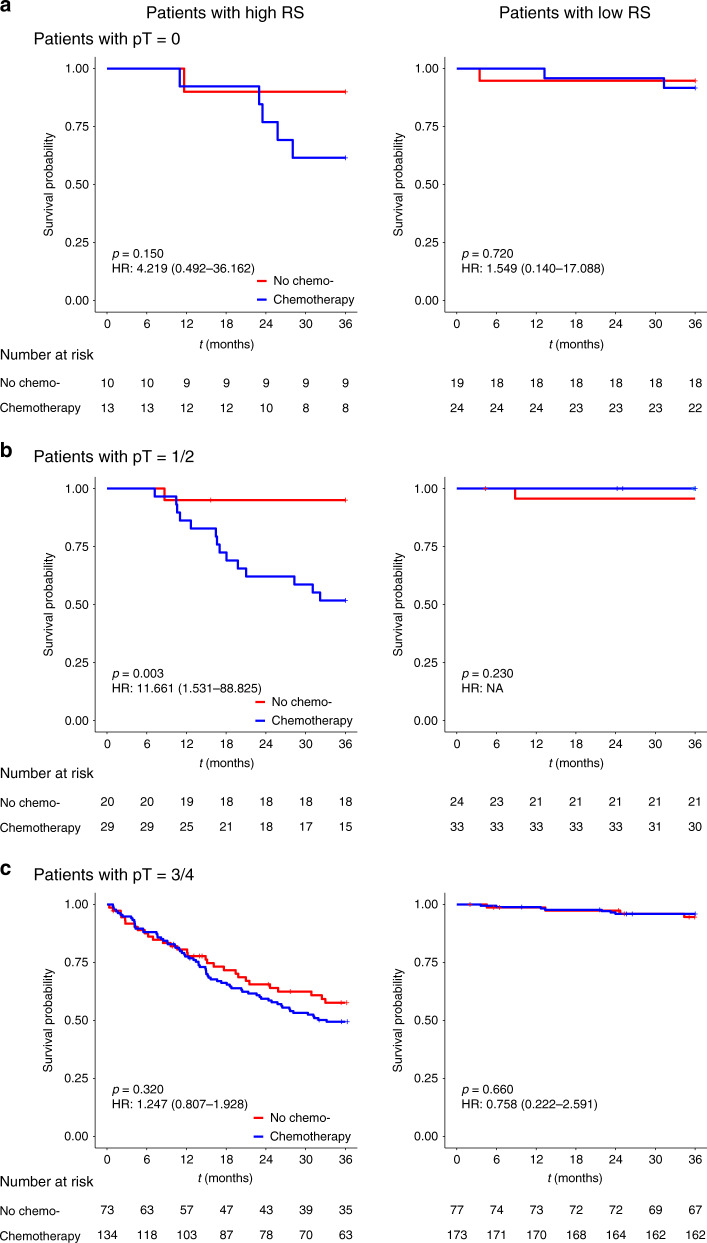
Adjuvant chemotherapy benefits based on DMFS according to pT stage and RS. **a**–**c** K–M DMFS curves are shown for patients according to their use of adjuvant chemotherapy. In addition, patients with a high RS (left) were stratified according to pT0 (*n* = 23, upper), pT1–2 (*n* = 49, middle), and pT3–4 (*n* = 207, bottom). Patients with a low RS (right) were also stratified according to pT0 (*n* = 43, upper), pT1–2 (*n* = 57, middle), and pT3–4 (*n* = 250, bottom). *p* values were calculated using two-sided log-rank test. RS radiomic signature; HR hazard ratio; DMFS distant metastasis-free survival; LARC locally advanced rectal cancer. Source data are provided as a Source Data file.

**Fig. 6:**
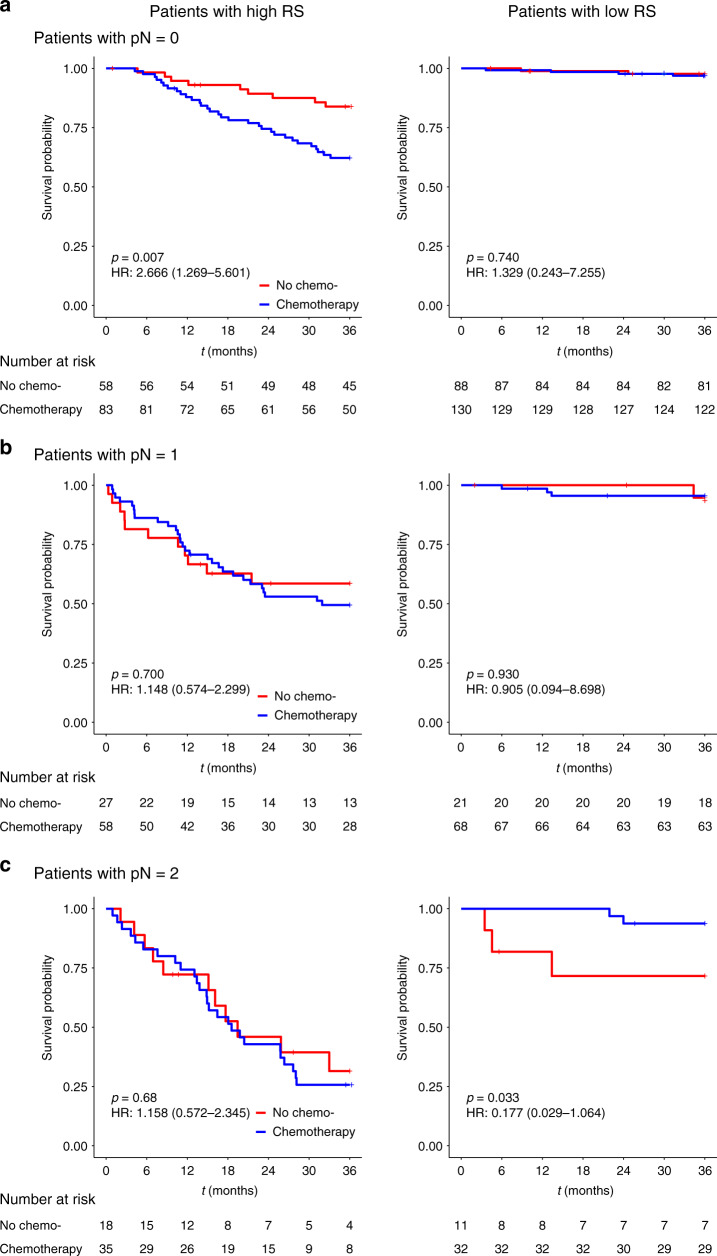
Adjuvant chemotherapy benefits based on DMFS according to pN stage and RS. **a**–**c** K–M DMFS curves are shown for patients according to their use of adjuvant chemotherapy. In addition, patients with a high RS (left) were stratified according to pN0 (*n* = 141, upper), pN1 (*n* = 85, middle), and pN2 (*n* = 53, bottom). Patients with a low RS (right) were also stratified according to pN0 (*n* = 218, upper), pN1 (*n* = 89, middle), and pN2 (*n* = 43, bottom). *p* values were calculated using two-sided log-rank test. RS radiomic signature; HR hazard ratio; DMFS distant metastasis-free survival; LARC locally advanced rectal cancer. Source data are provided as a Source Data file.

The interaction tests for pathological stage and adjuvant chemotherapy efficacy in the high- and low-radiomic signature groups were also performed. The results for pT stage subgroup analysis indicated that, in the high-radiomic signature group, pT1–2 patients did not benefit from the adjuvant chemotherapy (HR: 11.661, 95% CI: 1.531–88.825, *p* = 0.003; *p* < 0.001 for interaction), while no significant interactions were observed in the low-radiomic signature group (Fig. 5). The results for pN stage subgroup analysis indicated that, pN0 patients with high-radiomic signature and adjuvant chemotherapy, had even worse survival than those with high-radiomic signature but without adjuvant chemotherapy (HR: 2.666, 95% CI: 1.269–5.601, *p* = 0.007; *p* < 0.001 for interaction), while in the low-radiomic signature group, only pN2 patients had survival benefit from the adjuvant chemotherapy (HR: 0.177, 95% CI: 0.029–1.064, *p* = 0.033; *p* < 0.001 for interaction) (Fig. 6).

These results of interaction tests suggest that not all LARC patients will benefit from adjuvant chemotherapy, and the treatment strategy should be carefully selected based on the pathological stage and radiomic signature as well.

## Discussion

This study not only developed and validated a radiomic signature with a series of comprehensive MRI features associated with prognosis of LARC patients, but also investigated the association between the radiomic signature and chemotherapy efficacy. The proposed radiomic signature was able to predict DM better than traditional clinicopathological characteristics. More importantly, stratified with the radiomic signature and pathological stage, patients that can benefit from adjuvant chemotherapy could be identified.

There is broad variability in the outcomes among LARC patients, even patients with the same disease stage, which makes accurate prognostication essential for treatment planning^23^. Previous studies have revealed the prognostic value of radiomic features in rectal cancer^24–26^, and we provide further evidence from a multicenter study that a radiomic signature could independently predict DMFS. Furthermore, combining the radiomic signature with clinicopathological information in a radiomic nomogram had significantly better ability to predict DM than a clinical nomogram and the previously proposed VN, based on larger C-index values in all four cohorts. In addition, the generalizability of the radiomic nomogram was better than that of the clinical model and the VN. This may be because clinicopathological factors only reflect specific tumor characteristics, while radiomics based on multiparametric MRI can comprehensively and quantifiably characterize the tumor phenotype^20^. It is also possible that high-dimensional imaging features provide additional information, allowing radiomics to be less affected by patient distributions in the different cohorts, which might explain its better generalizability.

The radiomic signature’s good ability to predict DM confirms its prognostic value, which could be used to stratify patients into groups with high and low risks of DM. This approach might allow clinicians to select more personalized and hopefully more effective treatment strategies. Furthermore, when we considered clinical stage with the radiomic signature, we were also able to identify patient groups with different risks of DM in all four cohorts. This result suggests that the radiomic signature could also help guide personalized treatment of patients with the same clinical stage. Thus, the radiomic nomogram that combines the radiomic signature with traditional clinicopathological information might be a useful prognostic tool for clinicians. This nomogram contained improved the prognostic ability of the clinical staging system, and could be developed as an easy-to-use tool. In addition, the clinical utility of radiomic nomogram was assessed using decision curve analysis, which is commonly used method in radiomic studies^27,28^, and the results suggest that the radiomic nomogram could benefit patients.

The most important finding of this study was that stratified with radiomic signature and pathological stage, patients with pN2 disease in the low-radiomic signature group experienced a substantial benefit from chemotherapy, and in contrast, for patients with pT1–2 or pN0 disease in the high-radiomic signature, receiving adjuvant chemotherapy may indicate worse prognosis compared with not receiving adjuvant chemotherapy. Although the current guidelines recommend adjuvant chemotherapy for most LARC patients, some studies have plausibly found that not all patients will benefit from chemotherapy^7,29^. Therefore, it can be useful to be able to personalize the chemotherapy or treatment strategy to improve patient outcomes. Previous studies have developed valuable radiomic models to identify patients with various cancers who will benefit from different therapies, based on findings from CT, MRI, and PET-CT^30–32^. Our findings are consistent with previous reports that chemotherapy was unnecessary for patients with pN0 disease^6^ or patients who achieve pCR^7,29^, and suggest that more aggressive systemic therapy should be considered in these cases. Thus, the radiomic signature may be useful for identifying patients who should and should not undergo chemotherapy in this setting.

The clinical advantages of evaluating the radiomic signature are that it is non-invasive and can be repeated at different disease states. Moreover, the extraction of quantitative MRI features provides high-dimensional description of the intra-tumor heterogeneity. Interestingly, the MRI sequence appears to be important, as the features of the radiomic signature were all from apparent diffusion coefficient (ADC) maps (calculated with DWI). This finding is consistent with our previous findings, which indicated that ADC maps were valuable for evaluating the effects of neoadjuvant therapy in rectal cancer^17^ and breast cancer^18^, which would suggest that the radiomic signature is a fairly reliable marker.

The present study has some limitations that merit consideration. The first is the limited sample size and retrospective data collection, which suggest that the model should be validated in larger well-designed prospective studies. The accumulation of additional patients will also allow for the collection of more patient- and tumor-specific information, which can be used to construct a more stable and accurate model. Second, while imaging features focus on the macro tumor information, it would be interesting to examine whether digital biopsy, pathological imaging, and genomic sequencing may provide more micro information. Third, we only examined the performance of the radiomic signature in Chinese patients, and it remains unclear whether it will perform to the same level in different ethnic populations, which could be worthy for future studies.

In conclusion, we identified a multiparametric MRI-based radiomic signature that effectively predicted DMFS in LARC patients and improved the performance of the traditional clinicopathological prediction model. Combining the radiomic signature with pathological stage might help identify which patients are expected to benefit from adjuvant chemotherapy.

## Methods

### Patients

This retrospective multicenter study was conducted in accordance with the Declaration of Helsinki. The study’s protocol was approved by the ethics committee of each participating hospital. All procedures followed the approved protocol and the requirement for informed consent was waived.

A total of 629 consecutive LARC patients were included at 5 hospitals selected from different regions of China. The detailed inclusion and exclusion criteria are shown in Supplementary Methods and Supplementary Fig. 5, and the patients’ baseline characteristics were collected from their medical records (Table 1). The primary outcome was DMFS, which was defined as the time from surgery to the first confirmed instance of DM or death caused by disease or treatment. The minimum follow-up period was 36 months after surgery. Patients who were alive and free from disease (or died because of an unrelated cause and without evidence of rectal cancer) were censored at the last follow-up. All patients were postoperatively followed every 3–6 months during the first 2 years, every 6 months during the next 3 years, and then annually thereafter. The clinical evaluations included physical examination, measurement of serum carcinoembryonic antigen (CEA) level, imaging, and colonoscopy. CEA levels were tested at 3–6-month intervals for the first 2 years and at 6-month intervals for >2–5 years. Imaging, including contrast-enhanced computed tomography (CT) of the abdomen and pelvis, and unenhanced CT of the chest, was performed at a minimum of every 12 months and for at least three years. Colonoscopy was performed one year after surgery and then repeated every 2–5 years unless advanced adenomas were identified. All instances of DM were confirmed via histology or imaging.

The patients were divided into four cohorts (Supplementary Fig. 5): the primary cohort (*n* = 176 from centers 1 and 2) and three external validation cohorts (validation cohort 1: *n* = 154 from center 3, validation cohort 2: *n* = 150 from center 4, and validation cohort 3: *n* = 149 from center 5). The sample size evaluation is shown in Supplementary Methods.

### MRI data acquisition and imaging feature detection

All patients underwent MRI examination within 1 week before colonoscopy. To reduce colonic motility, 20 mg of scopolamine butyl bromide was injected intramuscularly 30 min before the MRI scan, although patients were not required to undergo bowel preparation before the examination. All patients underwent a conventional rectal MRI protocol that included DWI and T2WI. The DWI images were obtained using single-shot echo-planar imaging with two *b* values (0 and 1000 s/mm^2^). ADC maps were generated automatically and included both b values in a monoexponential decay model. The detailed MRI parameters at the five hospitals are shown in the Supplementary Table 3.

Each patient’s MRI data were collated for tumor masking and feature extraction. The regions of interest (ROIs) were delineated manually using the itk-SNAP software (www.itksnap.org) on each slice obtained via T2WI and DWI (delineated with *b* value of 1000 s/mm^2^ and then copied to the corresponding ADC maps). The procedures for tumor masking and evaluating inter-/intra-observer reproducibility are shown in Supplementary Methods.

Radiomic feature extraction was performed for each MRI scan with manually segmented ROIs, using an in-house toolbox developed with MATLAB 2016b (Mathworks, Natick, MA, USA). All images of each MRI scan for each patient was normalized separately using *Z*-scores to obtain a standard normal distribution of image intensities. Four groups of imaging features were then extracted: Group 1 had eight shape- and size-based features, Group 2 had 15 first-order statistical features, Group 3 had 53 textural features, and Group 4 had 544 wavelet features. The final feature set included 620 features for each MR sequence (T2WI and ADC), which corresponded to a total of 1240 radiomic features for each patient. Detailed information regarding the feature-extracting algorithms is provided in Supplementary Methods.

### Radiomic signature construction and validation

The radiomic signature was created with multiparametric MRI (T2WI and ADC) based on the primary cohort. The imaging features were first normalized (details are shown in Supplementary Methods), and then a coarse-to-fine feature selection strategy was used to reduce the risk of bias and potential overfitting. Univariate Cox analysis was initially used to detect the associations between each feature and the patients’ DMFS. All features were then ranked in ascending order according to the Cox *p* values, and the top 20% of the features with *p* < 0.1 were used for further analysis. Among these features, the Pearson correlation coefficients for each feature pair were then calculated (denoted as “*r*” hereafter). Feature pairs with |*r*| > 0.6 were selected, and then in each of these pairs, the feature with larger mean absolute correlation was removed. Finally, the LASSO algorithm with Cox analysis^19,31^ was used to identify the most useful prognostic features for constructing the radiomic signature.

The potential association between the radiomic signature and DMFS was initially assessed in the primary cohort and then validated in the validation cohorts based on Kaplan–Meier survival analysis. The median value for the radiomic signature in the primary cohort was used as the cutoff for dividing patients into groups with high or low-radiomic signatures. The same cutoff value was applied to all the validation cohorts. The prognostic accuracy of the radiomic signature for patient stratification was assessed in the primary and validation cohorts using time-dependent receiver operating characteristic (ROC) curve analysis. The ROC curves for 1-year, 2-year and 3-year DMFS were plotted for all cohorts, and the AUCs were quantified. Kaplan–Meier survival analysis was also performed to explore whether the radiomic signature was associated with DMFS within clinical and pathological stage subgroups for each cohort.

### Assessing the incremental value of radiomic signature

We also evaluated a clinicopathologic model based on 15 risk factors and a radiomic nomogram to determine whether the radiomic signature added incremental value for predicting DM in LARC patients. These models were tested in the primary and validation cohorts. We also evaluated the performance of VN^9^. Detailed information regarding these models is provided in Supplementary Methods. The radiomic nomogram’s performance was evaluated based on Harrell’s concordance index (C-index), calibration curves and decision curve analysis. The NRI^33^ and IDI^34^ values were evaluated to quantify the radiomic signature’s incremental prognostic improvement.

### Radiomic signature and chemotherapy

Radiomic features are associated with the effects of anti-tumor therapy in different cancers^27,30,32^. Therefore, we explored the potential association between the radiomic signature and chemotherapy efficacy among all patients (based on DMFS). Furthermore, we examined the potential interaction between the radiomic signature and chemotherapy according to the high- and low-signature grouping. Stratified analyses were also performed according to the clinical factors and radiomic signature level associated with chemotherapy efficacy, in order to identify patient subgroups that could benefit from adjuvant chemotherapy. Interaction tests for the radiomic signature, clinical factors, and chemotherapy were also performed.

### Statistics and reproducibility

Intergroup comparisons were performed using the *t* test or Mann–Whitney *U* test for continuous variables, and using the *Χ*^2^ test or Fisher’s test for categorical variables, as appropriate. All tests were two-sided and results were considered significant at *p* < 0.05. R software was used for model building (version 3.5.2; https://www.r-project.org/). The packages used in the current study included glmnet, timeROC, rms, survival, Hmisc, nricens, and PredictABEL. All statistical analyses were performed using IBM SPSS software (version 21; IBM Corp, Armonk, NY, USA).

### Reporting summary

Further information on research design is available in the Nature Research Reporting Summary linked to this article.

## Supplementary information

Supplementary Information

Reporting Summary

## Data Availability

The source data underlying Figs. 1, 2, 4, 6, Supplementary Figs. S2, S3, S5, and Table 2 is provided as a Source Data file. The MRI imaging data and clinical information, analyzed during the current study are not publicly available for patient privacy purposes, but are available from the corresponding author J.T. upon reasonable request. All the other data supporting the findings of this study are available within the article and its supplementary information files.

## Code availability

Source code of proposed method can be found in the following Github repository, https://github.com/SK94-ai/Radiomics-Predicting-Distant-Metastasi.
