# Supplementary material for: Predicting distant metastasis and chemotherapy benefit in locally advanced rectal cancer
Source: Nat Commun. 2020 Aug 27;11:4308. doi: 10.1038/s41467-020-18162-9 (PMC7452897; doi:10.1038/s41467-020-18162-9)
Supplement: Supplementary file 1 — Supplementary Information [file 41467_2020_18162_MOESM1_ESM.pdf]

SUPPLEMENTARY INFORMATION:

Predicting Distant Metastasis and Chemotherapy Benefit in Locally Advanced Rectal Cancer

Liu et al.

Supplementary Methods

All patients were enrolled with strict inclusion and exclusion criteria, which are shown as follows:

Inclusion criteria

a) Primary rectal adenocarcinoma confirmed via biopsy;
b) Locally advanced disease (stage II or III) determined tumor based on pre-treatment computed tomography of the chest and abdomen and pelvis magnetic resonance imaging, according to the 8th edition of the AJCC Staging Manual;
c) A tumor that was located within 12 cm of the anal verge;
d) Age of 18-75 years;
e) No other treatment before the MRI scan;
f) MRI scan including diffusion-weighted MRI, T2-weighted MRI;
g) Radical surgery was performed.

Exclusion criteria

a) Patients with a history of cancer <5 years;
b) Patients with residual tumor and/or circumferential resection margin involvement;
c) Patients with <3 years of follow-up data;
d) Patients who did not undergo postoperative imaging or clinical follow-up to detect recurrence.

Following baseline clinical information for each patient were analyzed in this study:

a) Clinical characteristics, including sex, age at surgery, clinical stage (c stage), clinical tumor (cT) stage, clinical nodal (cN) stage and tumor location. Tumor location was categorized based on distance from the anorectal verge: <5 cm, 5-10 cm, and >10 cm.
b) Treatment factors, including preoperative therapy protocol (yes or no), surgical procedure (Dixon and preventive ileostomy, Dixon, Miles, or Hartmann), surgical approach (laparoscopic resection or open resection), and fluorouracil-based adjuvant chemotherapy (yes or no), postoperative radiotherapy (yes or no).
c) Pathological factors, including tumor stage (pT) and nodal stage (pN). The pathological factors were judged based on the 8th edition of the AJCC Cancer Staging Manual and the NCCN guidelines.

d) Preoperative serum CEA level. The preoperative CEA level was tested within one week before surgery. The CEA levels were categorized as normal (≤5.0 ng/mL) or elevated (>5.0 ng/mL).

In total, 629 patients with complete clinical information were recruited from the five centers according to the inclusion and exclusion criteria. Details about the inclusion and exclusion of patients from each hospital and division of primary and validation cohorts were shown in Supplementary Figure 1.

Sample size evaluation

In this study, the proportional hazards regression model was used for the prognosis of distant metastasis free survival of the patients. To avoid potential overfitting of the model, the minimum sample size for the primary and validation cohorts was estimated.

For the sample size evaluation of the primary cohort, the rule of thumb is that the number of predictors should remain within 1/15–1/10 of the sample size in the training dataset (i.e., the primary cohort) (1, 2). In this study, 4 imaging features were selected for radiomic signature, while the sample size of the primary cohort was 176. Thus, the relative between the number of patients in the primary cohort and the selected features for the radiomic signature was acceptable.

For the sample size evaluation of the validation cohort, a sample size estimation method proposed in the book "Sample Size Calculations in Clinical Research 2nd Ed" was performed. According to the book, the sample size calculation could be estimated using following formula:

Letting θ represent the hazard ratio, the hypotheses of interest are:

$$H_0: \theta = \theta_0$$

$$H_1: \theta \neq \theta_0$$

The sample size and power are calculated respectively:

$$n = \frac{1}{\sigma^2 p_E}\left(\frac{z_{1-\alpha/2}+z_{1-\beta}}{\ln(\theta)-\ln(\theta_0)}\right)^2 \qquad (1)$$

$$1-\beta = \Phi\left(z - z_{1-\alpha/2}\right) + \Phi\left(-z - z_{1-\alpha/2}\right),\ z = (\ln(\theta)-\ln(\theta_0))\sigma\sqrt{np_E} \qquad (2)$$

where, n is the sample size for model validation, Φ is the standard Normal distribution function, α is Type I error, β is Type II error, $1-\beta$ is power, θ_0 is the hazard ratio hypothesized under the null hypothesis, θ is the hazard ratio, p_E is the overall probability of the event occurred, and σ^2 is the variance of the covariate.

In our study, the overall probability of the event (DM ratio) in 3 years occurred in the pilot experiment was 0.29 (p_E, 51/176, data in the primary cohort is used as a pilot experiment), the log hazards ratio associated with a one-unit change and the variance of radiomic signature were 6.628 ($\ln(\theta)$) and 0.801 (σ^2), respectively. $\ln(\theta_0)$ was set to 0 for the null hypothesis. The results showed that the sample size needed in the validation cohort was calculated to be 16 with the desired two-sided significance level $\alpha = 0.05$ and power $1 - \beta = 95\%$, referring to the primary cohort. In this study, 154, 150, and 149 patients were enrolled into the three validation cohorts, respectively.

In conclusion, both the sample size for primary and validation cohorts in this study was enough.

Tumor masking

Five radiologists (1 from each participating hospital) with at least 10 years' experience in rectal MR imaging were chiefly responsible for the evaluation of tumor masking. ROIs were drawn along the contour of the tumor as visualized by T2WI (slightly high signal), containing the surrounding chords and burrs. ROIs were placed on the high signal intensity region on DWI on each slice. Due to the higher resolution of DWI compared with ADC maps, ROIs were detected with a b-value of 1,000 s/mm^2 first and then copied to the corresponding ADC maps for further analysis. Inter- and intra-observer reproducibility of tumor masking and radiomic feature extraction were initially analyzed with the T2WI data of 50 randomly selected patients for ROI-based radiomic feature generation in a blinded fashion by these 5 radiologists.

To ensure reproducibility, each radiologist repeated the tumor masking and generation of radiomic features twice with an interval of at least 1 month, following the same procedure. Intra-class correlation coefficients (ICCs) were utilized for evaluating the intra- and inter-observer agreement in terms of feature extraction. We interpreted an ICC of 0.81-1.00 as almost perfect agreement, 0.61-0.80 as substantial agreement, 0.41-0.60 as moderate agreement, 0.21-0.40 as fair agreement, and 0-0.20 as poor or no agreement. An ICC greater than 0.6 was considered a mark of satisfactory inter- and intra-observer reproducibility.

To ensure the accuracy of tumor masking, the tumor masks were evaluated by other radiologists from the same hospital for each hospital, following the same guideline describing how to define the boundary of tumors.

Radiomic feature extraction

Four groups of imaging features were extracted from both T2 images and ADC maps: Group 1 with 8 shape- and size-based features, Group 2 with 15 first order statistical features, Group 3 with 53 textural features, Group 4 with 544 wavelet features. Following showed the details of the features:

(1) Shape and size-based features

In this group of features, we included descriptors of the three-dimensional shape- and size of the tumor region. Let in the following definitions V denote the volume and A the surface area of the volume of interest. We determined the following shape and size-based features:

1. **Compactness 1:**

$$\frac{V}{\sqrt{\pi}A^{\frac{2}{3}}} \tag{3}$$

2. **Compactness 2:**

$$36\pi\frac{V^2}{A^3} \tag{4}$$

3. **Maximum 3d diameter:**

   The maximum three-dimensional tumor diameter is measured as the largest pairwise Euclidean distance, between voxels on the surface of the tumor volume.

4. **Spherical disproportion:**

$$\frac{A}{4\pi R^2} \tag{5}$$

5. **Sphericity:**

$$\frac{\pi^{\frac{1}{3}}(6V)^{\frac{2}{3}}}{A} \tag{6}$$

6. **Surface area:**

   The surface area is calculated by triangulation (i.e. dividing the surface into connected triangles) and is defined as:

$$A=\sum_{i=1}^{N}\frac{1}{2}\left|\mathbf{a}_i\mathbf{b}_i\times\mathbf{a}_i\mathbf{c}_i\right| \tag{7}$$

   Where N is the total number of triangles covering the surface and a, b and c are edge vectors of the triangles.

7. **Surface to volume ratio:**

$$\frac{A}{V} \tag{8}$$

8. Volume:

The volume (V) of the tumor is determined by counting the number of pixels in the tumor region and multiplying this value by the voxel size.

(2) First order statistical features

The following 15 statistical features were extracted.

Let X be the three-dimensional image matrix with N voxels of the ROI and P be the first order histogram distribution with N_g discrete intensity levels.

1. IntensityMax: The maximum intensity value of X.
2. IntensityMin: The minimum intensity value of X.
3. Median: The median intensity value of X.
4. IntensityStd:

$$\left(\frac{1}{N-1}\sum_{i=1}^{N}(\mathbf{X}(i)-\bar{\mathbf{X}})^2\right)^{1/2} \tag{9}$$

5. Mean:

$$\frac{1}{N}\sum_{i}^{N}\mathbf{X}(i) \tag{10}$$

6. Variance:

$$\frac{1}{N-1}\sum_{i=1}^{N}(\mathbf{X}(i)-\bar{\mathbf{X}})^2 \tag{11}$$

Where $\bar{X}$ is the mean of X.

7. Range:

The range of intensity values of X.

8. Mean absolute deviation:

The mean of the absolute deviations of all voxel intensities around the mean intensity value

9. Energy:

$$\sum_{i}^{N}\mathbf{X}(i)^2 \tag{12}$$

10. Entropy:

$$\sum_{i=1}^{N_g} \mathbf{P}(i) \log_2 \mathbf{P}(i) \tag{13}$$

11. Entropy_p:

$$\sum_{i=1}^{N_g} \frac{\mathbf{P}(i)}{N} \log_2 \frac{\mathbf{P}(i)}{N} \tag{14}$$

12. Root mean square:

$$\sqrt{\frac{\sum_{i}^{N} \mathbf{X}(i)^2}{N}} \tag{15}$$

13. Uniformity:

$$\sum_{i=1}^{N_g} \mathbf{P}(i)^2 \tag{16}$$

14. Uniformity_p:

$$\sum_{i=1}^{N_g} (\frac{\mathbf{P}(i)}{N})^2 \tag{17}$$

15. Mass:

The sum intensity value of X.

Textural features

Second order statistic texture features, and higher order statistic texture features were extracted. Twenty-two second order statistic texture features could be calculated from the Gray Level Co-occurrence Matrix (GLCM). Thirty-one high order statistic texture features were calculated from the Gray Level Size Zone Matrix (GLSZM), Gray Level Run Length Matrix (GLRLM), and Neighborhood Gray Tone Difference Matrix (NGTDM).

Gray-Level Co-Occurrence Matrix based features (GLCM)

GLCM based features were second-order statistical texture features, which are defined as a matrix M (i, j; δ, θ) to indicate the relative frequency with intensity values of pixels (i and j) at the distance of δ in direction θ. Texture matrices were determined considering 26 connected voxels (i.e. voxels were considered to be neighbors in all 13 directions in three dimensions). In this study, δ was set to 1 and θ to each of the 13 directions in three dimensions, yielding a total of 13 gray GLCM for each 3D image. From these GLCM matrices, 22 textural features are derived. Each 3D GLCM based feature was then calculated as the mean of the feature calculations for each of the 13 directions.

Let:

M (i, j) be the co-occurrence matrix for an arbitrary δ and θ, set δ=1 and θ=0 and 45,

N_g be the number of discrete intensity levels in the images, set as 25,

μ be the mean of M (i, j),

$\mathbf{m}_x(i) = \sum_{j=1}^{N_g} \mathbf{M}(i, j)$ be the marginal row probabilities,

$\mathbf{m}_y(i) = \sum_{i=1}^{N_g} \mathbf{M}(i, j)$ be the marginal column probabilities, and u_y ,μ_x, be the mean of m_x .and m_y

$$\mathrm{HX} = -\sum_{i=1}^{N_g} m_x(i)\log(m_x(i)), \quad (18)$$

$$\mathrm{HY} = -\sum_{i=1}^{N_g} m_y(i)\log(m_y(i)), \quad (19)$$

$$\mathrm{HXY} = -\sum_{i=1}^{N_g}\sum_{j=1}^{N_g} M(i,j)\log(M(i,j)), \quad (20)$$

$$\mathrm{HXY1} = -\sum_{i=1}^{N_g}\sum_{j=1}^{N_g} M(i,j)\log(m_x(i)m_y(j)). \quad (21)$$

$$\mathrm{HXY2} = -\sum_{i=1}^{N_g}\sum_{j=1}^{N_g} m_x(i)m_y(j)\log(m_x(i)m_y(j)). \quad (22)$$

1. Energy:

$$\sum_{i=1}^{N_g}\sum_{j=1}^{N_g}[\mathbf{M}(i, j)]^2 \quad (23)$$

2. Contrast:

$$\sum_{i=1}^{N_g}\sum_{j=1}^{N_g}(i-j)^2 * \mathbf{M}(i, j) \quad (24)$$

3. Entropy:

$$-\sum_{i=1}^{N_g}\sum_{j=1}^{N_g}\mathbf{M}(i, j) * \log_2 \mathbf{M}(i, j) \quad (25)$$

4. Homogeneity 1:

$$\sum_{i=1}^{N_g}\sum_{j=1}^{N_g}\frac{\mathbf{M}(i, j)}{1+|i-j|} \quad (26)$$

5. Homogeneity 2:

$$\sum_{i=1}^{N_g}\sum_{j=1}^{N_g}\frac{\mathbf{M}(i, j)}{1+|i-j|^2} \quad (27)$$

6. Correlation:

$$\frac{\sum_{i=1}^{N_g}\sum_{j=1}^{N_g} ij\mathbf{M}(i,j)-\mu_i(i)\mu_j(j)}{\sigma_x(i)\sigma_y(j)} \tag{28}$$

7. Covariance:

$$\sum_{i=1}^{N_g}\sum_{j=1}^{N_g} ij\mathbf{M}(i,j)-\mu_i(i)\mu_j(j) \tag{29}$$

8. Sum Average:

$$\sum_{i=2}^{2N_g} i\mathbf{M}_{x+y}(i) \tag{30}$$

9. Sum Entropy:

$$-\sum_{i=2}^{2N_g} \mathbf{M}_{x+y}(i)\log_2\left[\mathbf{M}_{x+y}(i)\right] \tag{31}$$

10. Dissimilarity:

$$\sum_{i=1}^{N_g}\sum_{j=1}^{N_g} |i-j|\,\mathbf{M}(i,j) \tag{32}$$

11. Inverse Variance:

$$\sum_{i=1}^{N_g}\sum_{j=1}^{N_g} \frac{\mathbf{M}(i,j)}{(i-j)^2} \text{ , i} \neq \text{j} \tag{33}$$

12. Autocorrelation:

$$\sum_{i=1}^{N_g}\sum_{j=1}^{N_g} ij\mathbf{M}(i,j) \tag{34}$$

13. Cluster Prominence

$$\sum_{i=1}^{N_g}\sum_{j=1}^{N_g} \left[i+j-u_x-u_y\right]^4 \mathbf{M}(i,j) \tag{35}$$

14. Cluster Shade

$$\sum_{i=1}^{N_g}\sum_{j=1}^{N_g} \left[i+j-u_x-u_y\right]^3 \mathbf{M}(i,j) \tag{36}$$

15. Cluster Tendency

$$\sum_{i=1}^{N_g}\sum_{j=1}^{N_g} \left[i+j-u_x-u_y\right]^2 \mathbf{M}(i,j) \tag{37}$$

16. Difference Entropy

$$\sum_{i=0}^{N_g-1} \mathbf{M}_{x-y}(i)\log_2\left[\mathbf{M}_{x-y}(i)\right] \quad (38)$$

17. Maximum Probability:

$$\max\{\mathbf{M}(i,j)\} \quad (39)$$

18. Sum variance

$$\sum_{i=2}^{2N_g}(i-SE)^2\mathbf{M}_{x+y}(i) \quad (40)$$

19. Informational measure of correlation 1 (IMC1):

$$\frac{\mathrm{HXY}-\mathrm{HXY1}}{\max\{\mathrm{HX}-\mathrm{HY}\}} \quad (41)$$

20. Informational measure of correlation 2 (IMC2):

$$\sqrt{1-\mathrm{e}^{-2(\mathrm{HXY2}-\mathrm{HXY})}} \quad (42)$$

21. Inverse Difference Moment Normalized (IDMN):

$$\sum_{i=1}^{N_g}\sum_{j=1}^{N_g}\frac{\mathbf{M}(i,j)}{1+(\frac{|i-j|^2}{N^2})} \quad (43)$$

22. Inverse Difference Normalized (IDN):

$$\sum_{i=1}^{N_g}\sum_{j=1}^{N_g}\frac{\mathbf{M}(i,j)}{1+(\frac{|i-j|}{N^2})} \quad (44)$$

Gray Level Run Length Matrix based features (GLRLM)

GLRLM based features were high-order statistical texture feature, which were defined as P(i, j; θ) to indicate the number of times j and gray level i appear consecutively in the direction θ. In this study, a GLRLM was computed for every of the 13 directions in three dimensions, from which the below textural features were derived. Each 3D GLRLM feature was then calculated as the mean of the feature values for each of the 13 directions.

Let:

P (i, j; θ) be the run-length matrix P for a direction θ, set θ=0 and 45

N_g be the number of discrete intensity values,

N_r be the number of different run lengths, and

N_p be the number of voxels in the ROI.

1. Short Run Emphasis (SRE):

$$\frac{\sum_{i=1}^{N_g}\sum_{j=1}^{N_r}[\frac{\mathbf{P}(i,j;\theta)}{j^2}]}{\sum_{i=1}^{N_g}\sum_{j=1}^{N_r}\mathbf{P}(i,j;\theta)} \tag{45}$$

2. Long Run Emphasis (LRE):

$$\frac{\sum_{i=1}^{N_g}\sum_{j=1}^{N_r} j^2\mathbf{P}(i,j;\theta)}{\sum_{i=1}^{N_g}\sum_{j=1}^{N_r}\mathbf{P}(i,j;\theta)} \tag{46}$$

3. Gray-Level Nonuniformity (GLN):

$$\frac{\sum_{i=1}^{N_g}[\sum_{j=1}^{N_r}\mathbf{P}(i,j;\theta)]^2}{\sum_{i=1}^{N_g}\sum_{j=1}^{N_r}\mathbf{P}(i,j;\theta)} \tag{47}$$

4. Run-Length Nonuniformity (RLN):

$$\frac{\sum_{j=1}^{N_r}[\sum_{i=1}^{N_g}\mathbf{P}(i,j;\theta)]^2}{\sum_{i=1}^{N_g}\sum_{j=1}^{N_r}\mathbf{P}(i,j;\theta)} \tag{48}$$

5. Run Percentage (RP):

$$\sum_{i=1}^{N_g}\sum_{j=1}^{N_r}\frac{\mathbf{P}(i,j;\theta)}{N_p} \tag{49}$$

6. Low Gray-Level Run Emphasis (LGRE):

$$\frac{\sum_{i=1}^{N_g}\sum_{j=1}^{N_r}[\frac{\mathbf{P}(i,j;\theta)}{i^2}]}{\sum_{i=1}^{N_g}\sum_{j=1}^{N_r}\mathbf{P}(i,j;\theta)} \tag{50}$$

7. High Gray-Level Run Emphasis (HGRE):

$$\frac{\sum_{i=1}^{N_g}\sum_{j=1}^{N_r} i^2 \mathbf{P}(i,j;\theta)}{\sum_{i=1}^{N_g}\sum_{j=1}^{N_r} \mathbf{P}(i,j;\theta)} \tag{51}$$

8. Short Run Low Gray-Level Emphasis (SRLGE):

$$\frac{\sum_{i=1}^{N_g}\sum_{j=1}^{N_r} \frac{\mathbf{P}(i,j;\theta)}{i^2 j^2}}{\sum_{i=1}^{N_g}\sum_{j=1}^{N_r} \mathbf{P}(i,j;\theta)} \tag{52}$$

9. Short Run High Gray-Level Emphasis (SRHGE):

$$\frac{\sum_{i=1}^{N_g}\sum_{j=1}^{N_r} \frac{i^2\mathbf{P}(i,j;\theta)}{j^2}}{\sum_{i=1}^{N_g}\sum_{j=1}^{N_r} \mathbf{P}(i,j;\theta)} \tag{53}$$

10. Long Run Low Gray-Level Emphasis (LRLGE):

$$\frac{\sum_{i=1}^{N_g}\sum_{j=1}^{N_r} \frac{j^2\mathbf{P}(i,j;\theta)}{i^2}}{\sum_{i=1}^{N_g}\sum_{j=1}^{N_r} \mathbf{P}(i,j;\theta)} \tag{54}$$

11. Long Run High Gray-Level Emphasis (LRHGE):

$$\frac{\sum_{i=1}^{N_g}\sum_{j=1}^{N_r} i^2 j^2 \mathbf{P}(i,j;\theta)}{\sum_{i=1}^{N_g}\sum_{j=1}^{N_r} \mathbf{P}(i,j;\theta)} \tag{55}$$

12. Mean:

$$\frac{1}{2N_{\mathrm{g}}}\sum_{i=1}^{N_g}\sum_{j=1}^{N_g} [\mathbf{P}(i,j)]^2 \tag{56}$$

13. Entropy:

$$\sum_{i=1}^{N_g}\sum_{j=1}^{N_g} \mathbf{P}(i,j) * \log_2 \mathbf{P}(i,j) \tag{57}$$

14. Energy:

$$\sum_{i=1}^{N_g}\sum_{j=1}^{N_g}[\mathbf{P}(i, j)]^2 \tag{58}$$

Gray Level Size Zone Matrix based features (GLSZM)

GLSZM based features were high-order statistical texture features, which were defined as P (i, j) to indicate the areas of size j and gray level i.

Let:

P (i, j) be the size zone of matrix P,

N_g be the number of discrete intensity values,

N_r be the number of different areas sizes,

N_p be the number of voxels in the ROI.

1. Small Zone Emphasis (SZE):

$$\frac{\sum_{i=1}^{N_g}\sum_{j=1}^{N_r}[\frac{\mathbf{P}(i, j)}{j^2}]}{\sum_{i=1}^{N_g}\sum_{j=1}^{N_r}\mathbf{P}(i, j)} \tag{59}$$

2. Large Zone Emphasis (LZE):

$$\frac{\sum_{i=1}^{N_g}\sum_{j=1}^{N_r} j^2\mathbf{P}(i, j)}{\sum_{i=1}^{N_g}\sum_{j=1}^{N_r}\mathbf{P}(i, j)} \tag{60}$$

3. Gray-Level Nonuniformity (GLN):

$$\frac{\sum_{i=1}^{N_g}[\sum_{j=1}^{N_r}\mathbf{P}(i, j)]^2}{\sum_{i=1}^{N_g}\sum_{j=1}^{N_r}\mathbf{P}(i, j)} \tag{61}$$

4. Zone-Size Nonuniformity (ZSN):

$$\frac{\sum_{j=1}^{N_r}[\sum_{i=1}^{N_g}\mathbf{P}(i, j)]^2}{\sum_{i=1}^{N_g}\sum_{j=1}^{N_r}\mathbf{P}(i, j)} \tag{62}$$

5. Zone Percentage (ZP):

$$\sum_{i=1}^{N_g}\sum_{j=1}^{N_r}\frac{\mathbf{P}(i,j)}{N_p} \tag{63}$$

6. Low Gray-Level Zone Emphasis (LGZE):

$$\frac{\sum_{i=1}^{N_g}\sum_{j=1}^{N_r}[\frac{\mathbf{P}(i,j)}{i^2}]}{\sum_{i=1}^{N_g}\sum_{j=1}^{N_r}\mathbf{P}(i,j)} \tag{64}$$

7. High Gray-Level Zone Emphasis (HGZE):

$$\frac{\sum_{i=1}^{N_g}\sum_{j=1}^{N_r}i^2\mathbf{P}(i,j)}{\sum_{i=1}^{N_g}\sum_{j=1}^{N_r}\mathbf{P}(i,j)} \tag{65}$$

8. Small Zone Low Gray-Level Emphasis (SZLGE):

$$\frac{\sum_{i=1}^{N_g}\sum_{j=1}^{N_r}\frac{\mathbf{P}(i,j)}{i^2 j^2}}{\sum_{i=1}^{N_g}\sum_{j=1}^{N_r}\mathbf{P}(i,j)} \tag{66}$$

9. Small Zone High Gray-Level Emphasis (SZHGE):

$$\frac{\sum_{i=1}^{N_g}\sum_{j=1}^{N_r}\frac{i^2\mathbf{P}(i,j)}{j^2}}{\sum_{i=1}^{N_g}\sum_{j=1}^{N_r}\mathbf{P}(i,j)} \tag{67}$$

10. Large Zone Low Gray-Level Emphasis (LZLGE):

$$\frac{\sum_{i=1}^{N_g}\sum_{j=1}^{N_r}\frac{j^2\mathbf{P}(i,j)}{i^2}}{\sum_{i=1}^{N_g}\sum_{j=1}^{N_r}\mathbf{P}(i,j)} \tag{68}$$

11. Large Zone High Gray-Level Emphasis (LZHGE):

$$\frac{\sum_{i=1}^{N_g}\sum_{j=1}^{N_r}i^2 j^2\mathbf{P}(i,j)}{\sum_{i=1}^{N_g}\sum_{j=1}^{N_r}\mathbf{P}(i,j)} \tag{69}$$

12. Gray-Level Variance (GLV):

$$\sum_{i=1}^{N_g}\sum_{j=1}^{N_r}\{i\mathbf{P}(i,j)-\frac{\sum_{i=1}^{N_g} i[\sum_{j=1}^{N_r}\mathbf{P}(i,j)]}{N_g N_r}\} \tag{70}$$

Neighborhood Gray Tone Difference Matrix based features (NGTDM)

NGTDM based features were high-order statistical texture features, which were defined as S(i) to indicate the sum of the absolute value between gray intensity level i and its neighbors' average intensity.

Let:

S(i) be the sum of absolute value between gray intensity level i and its neighbors' average intensity,

C(i) be the number of voxels with the gray intensity level i,

N_g be the number of discrete intensity values.

1. Coarseness:

$$\frac{1}{\varepsilon+\sum_{i=1}^{N_g}\frac{\mathbf{C}(i)\mathbf{S}(i)}{\sum_{i=1}^{N_g}\mathbf{C}(i)}} \tag{71}$$

2. Contrast:

$$\frac{\sum_{i=1}^{N_g}\sum_{j=1}^{N_g}\mathbf{C}(i)\mathbf{C}(j)(i-j)^2}{(\sum_{i=1}^{N_g}\mathbf{C}(i))^2}*\sum_{i=1}^{N_g}\mathbf{S}(i)*\frac{1}{N_g(N_g-1)\sum_{i=1}^{N_g}\mathbf{C}(\mathrm{i})} \tag{72}$$

3. Busyness:

$$\frac{\sum_{i=1}^{N_g}\mathbf{C}(i)\mathbf{S}(i)}{\sum_{i=1}^{N_g}\sum_{j=1}^{N_g}(i\mathbf{C}(i)-j\mathbf{C}(j))},\quad \mathbf{C}(i)\neq 0,\mathbf{C}(j)\neq 0 \tag{73}$$

4. Complexity:

$$\frac{1}{(\sum_{i=1}^{N_g}\mathbf{C}(i))^2}*\sum_{i=1}^{N_g}\sum_{j=1}^{N_g}\frac{|i-j|(\mathbf{C}(i)\mathbf{S}(i)+\mathbf{C}(j)\mathbf{S}(j))}{(\mathbf{C}(i)+\mathbf{C}(j))},\quad \mathbf{C}(i)\neq 0,\mathbf{C}(j)\neq 0 \tag{74}$$

5. Strength:

$$\frac{\sum_{i=1}^{N_g}\sum_{j=1}^{N_g}(\mathbf{C}(i)-\mathbf{C}(j))*(i-j)^2}{\sum_{i=1}^{N_g}\mathbf{C}(i)*\sum_{i=1}^{N_g}\mathbf{S}(i)}, \quad \mathbf{C}(i)\neq 0, \mathbf{C}(j)\neq 0 \tag{75}$$

(4) Wavelet features: first order statistical and texture features of a wavelet filtered image

Wavelet transform effectively decouples textural information by decomposing the original image into low- and high-frequencies. In this study, a 3D Coiflet wavelet transform was applied to each MR image, which decomposes the original image X into 8 decompositions. Consider L and H to be low-pass and high-pass function, the wavelet decompositions of X to be labeled as X_{LLL}, X_{LLH}, X_{LHL}, X_{LHH}, X_{HLL}, X_{HLH}, X_{HHL} and X_{HHH}. For example, X_{LLH} is then interpreted as the high-pass band, resulting from directional filtering of X with a low-pass filter along x-direction, a low pass filter along y-direction and a high-pass filter along z-direction and is constructed as:

$$\mathbf{X}_{\mathrm{LLH}}(i,j,k)=\sum_{p=1}^{N_L}\sum_{q=1}^{N_L}\sum_{r=1}^{N_H}\mathbf{L}(p)\mathbf{L}(q)\mathbf{L}(r)\mathbf{X}(i+p,j+q,k+r) \tag{76}$$

Where N_L is the length of filter L and N_H is the length of filter H. The other decompositions are constructed in a similar manner, applying their respective ordering of low or high-pass filtering in x, y and z-direction. A total of 544 (544 = (15 first order statistical features + 53 textural features) * 8) wavelet-based features were extracted for each modality.

Imaging feature normalization

Following normalization procedure was conducted to balance the deviance of radiomics features extracted from the four cohorts relying on different MRI protocols and environments and to generate appropriate input for further analysis.

First, all quantitative imaging features were normalized with a max-minimum normalization method in each cohort.

Second, a posteriori harmonization statistical method named Combat, which was proposed initially for genomics analysis to correct batch effect and applying in radiomic studies, was used for balancing the deviation of radiomic features extracted from the four cohorts. In this step, we first set the four cohorts as four batches, and then Combat was used.

Third, a method referred to inverse probability of treatment weighting (IPTW) was used to estimate the propensity scores. Specifically, principal component analysis (PCA) was applied to extract the main components of the radiomics features balanced by Combat, and 75% explained

components were used as the predictors to construct a logistic model for estimating the probability of a sample belonging to the primary cohort. The inverse of probabilities was used as the propensity scores. Here, DM patients and non-DM patients (the status at three years after surgery was used, without the censored data) were analyzed separately.

Finally, radiomic features were weighted based on the propensity scores to eliminate the covariable effect between the four cohorts.

Nomogram construction

To demonstrate the incremental value of the radiomics signature, a radiomic nomogram and two nomograms based on clinicopathologic risk factors were constructed and presented in validation cohorts.

For the nomogram based on the fifteen clinicopathologic risk factors (including age, sex, clinical stage, clinical T stage, lymph node status, location, neoadjuvant therapy, adjuvant chemotherapy, adjuvant radiotherapy, surgery procedure, surgery approach, pN stage, pT stage, LVI and PNI), univariate Cox analysis was initially used to detect the associations between each factor and the patients' DMFS, and the significant factors ($p<0.05$) were used to build a multivariate Cox model. Backward step-wise selection was applied using the likehood ratio test with Akaike information criterion employed as the stopping rule.

For the radiomic nomogram construction, multivariate Cox analysis was applied with radiomic signature and clinicopathologic risk factors that significantly related with the DMFS detected with univariate Cox analysis ($p<0.05$). Backward step-wise selection was applied using the likelihood ratio test with Akaike information criterion employed as the stopping rule.

In addition, we used the factors in nomogram for predicting distant metastases suggested by a previous paper published in Journal of Clinical Oncology and referred by the AJCC guideline (3) to constructed a clinical nomogram named VN with the primary cohort.

Formulas for nomograms:

Radiomic nomogram = 1.8098 * RS -0.7152 * surgery approach + 0.4962 * pN

Clinical nomogram = -0.8363 * surgery approach + 0.7523 * pN

VN = 0.7092×adjuvant chemotherapy +0.0993×surgery procedure +0.7157×pN +0.0602×pT

here, RS means radiomic signature, VN means Valentini's nomogram.

Supplementary Figures:

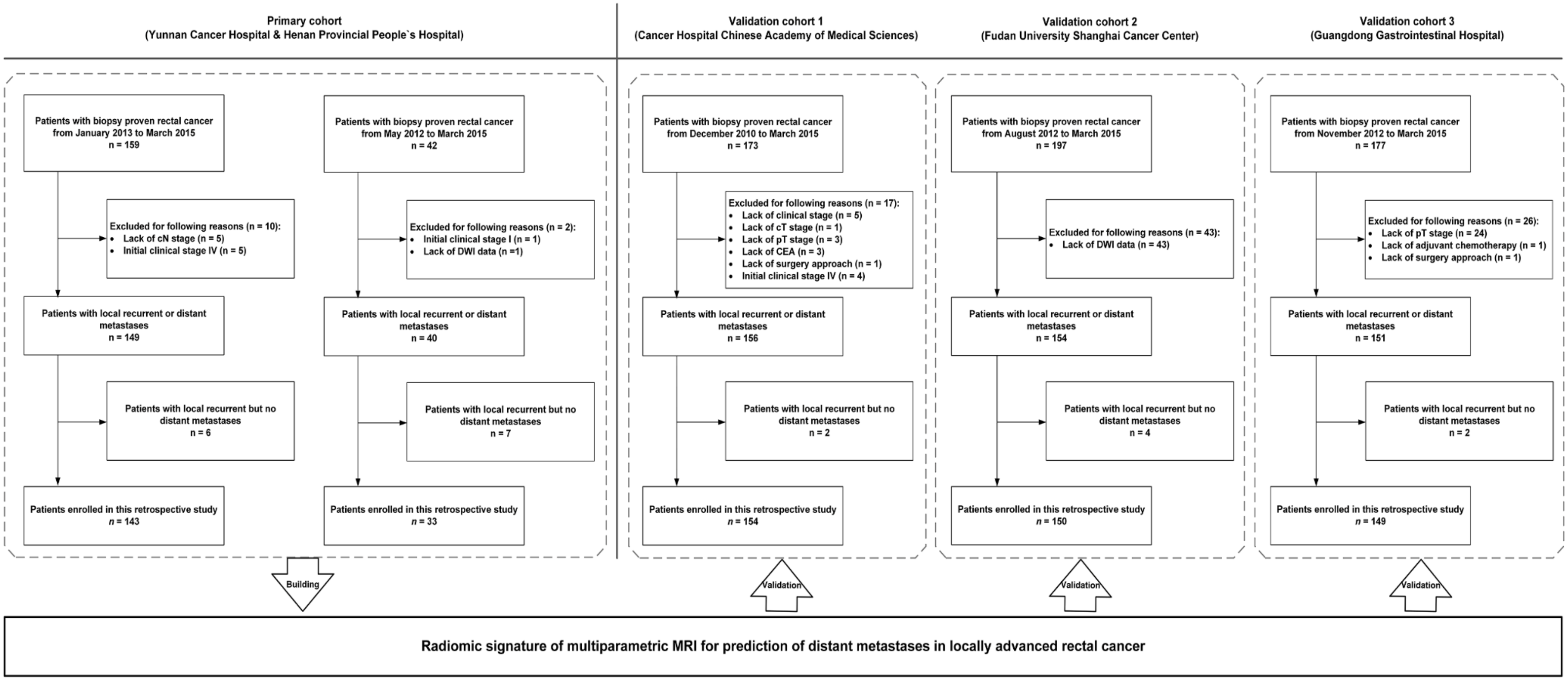

Supplementary Figure 1. Patient recruitment and study design.

This study included 629 of 748 patients at 5 Chinese hospitals, who were used to construct and validate the radiomic model.

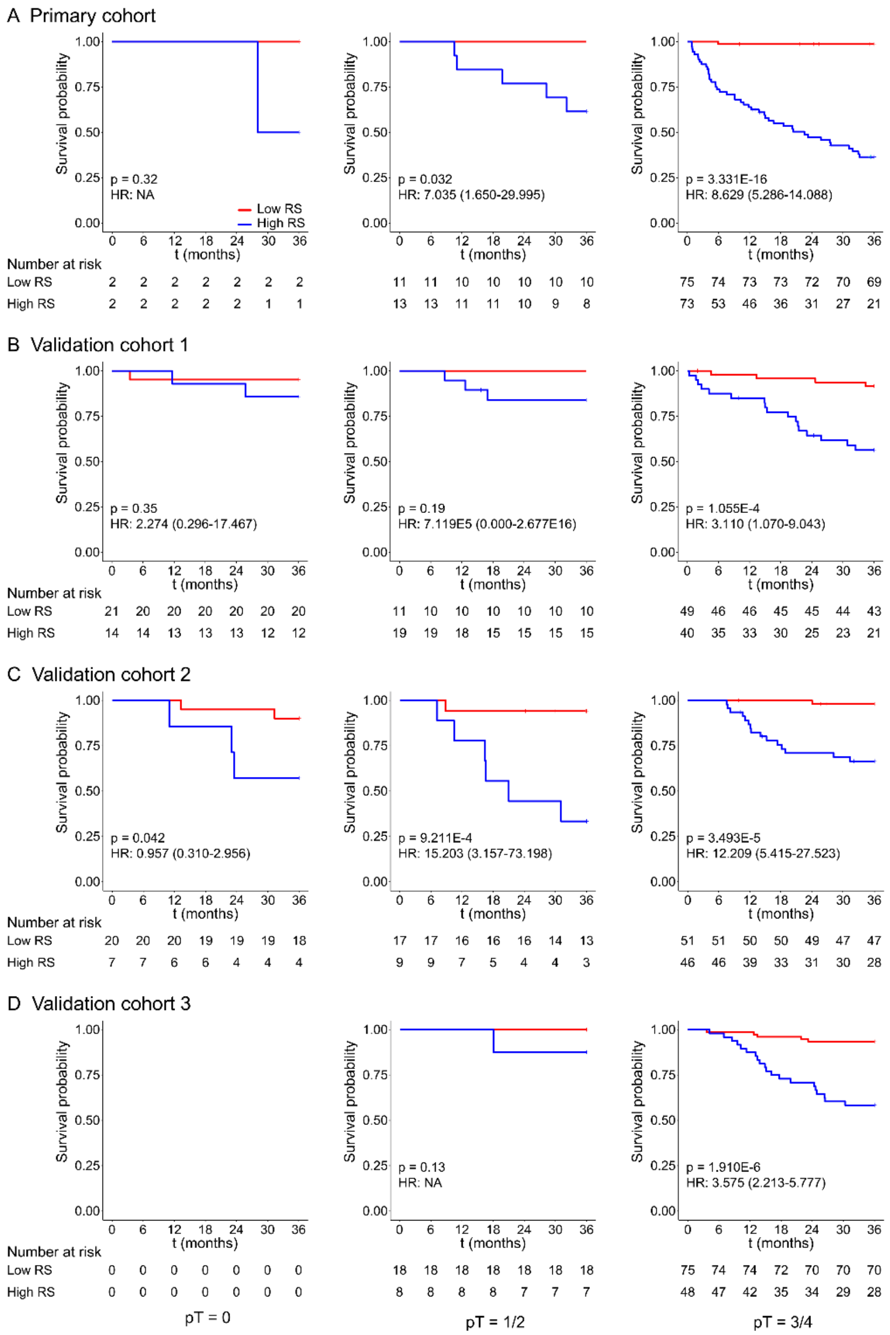

Supplementary Figure 2. Kaplan-Meier DMFS curves according to RS and pT stage among LARC patient subgroups in the primary and validation cohorts.
(A) The primary cohort (left: pT0, n = 4; middle: pT1–2, n = 24; and right: pT3–4, n = 148). (B) Validation cohort 1 (left: pT0, n = 35; middle: pT1–2, n = 30; and right: pT3–4, n = 89). (C) Validation cohort 2 (left: pT0, n = 27; middle: pT1–2, n = 26; and right: pT3–4, n = 97). (D) Validation cohort 3 (left: pT0, n = 0; middle: pT1–2, n = 26; and right: pT3–4, n = 123).
P-values were calculated using two-sided log-rank test. RS: radiomic signature; DMFS: distant metastasis-free survival; LARC: locally advanced rectal cancer.

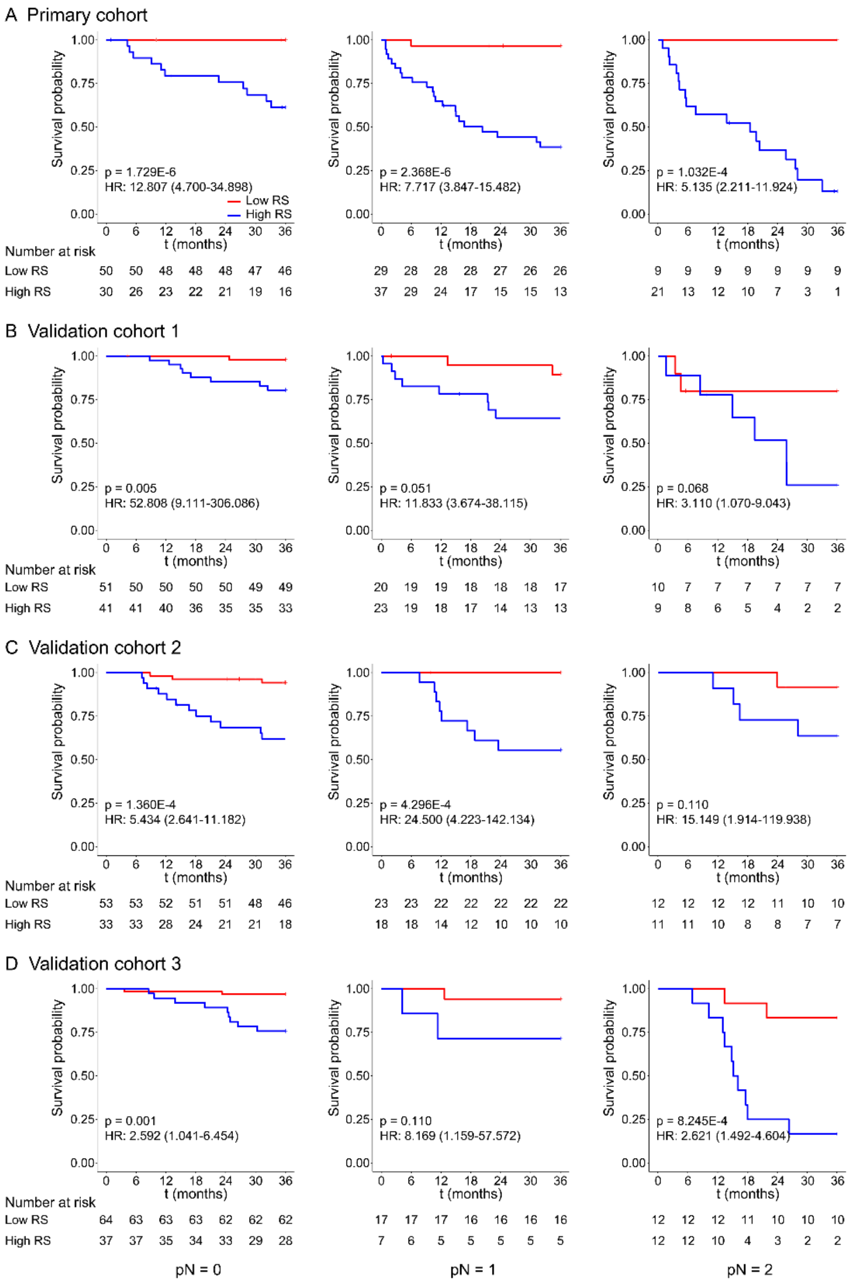

Supplementary Figure 3. Kaplan-Meier DMFS curves according to RS and pN stage among LARC patient subgroups in the primary and validation cohorts.

(A) The primary cohort (left: pN0, n = 80; middle: pN1, n = 66; and right: pN2, n = 30). (B) Validation cohort 1 (left: pN0, n = 92; middle: pN1, n = 43; and right: pN2, n = 19). (C) Validation cohort 2 (left: pN0, n = 86; middle: pN1, n = 41; and right: pN2, n = 23). (D) Validation cohort 3 (left: pN0, n = 101; middle: pN1, n = 24; and right: pN2, n = 24).

P-values were calculated using two-sided log-rank test. RS: radiomic signature; DMFS: distant metastasis-free survival; LARC: locally advanced rectal cancer.

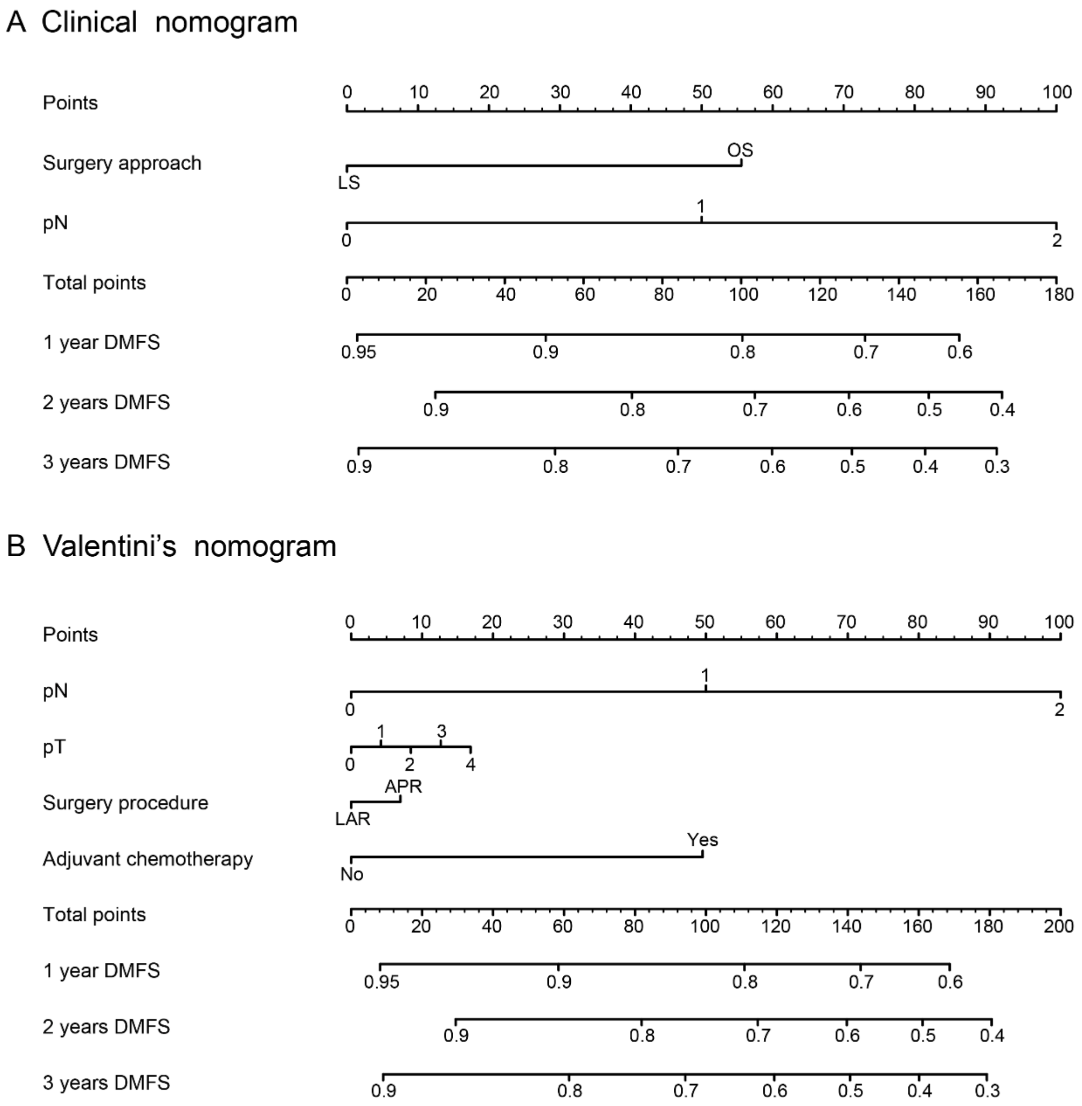

Supplementary Figure 4. Clinical nomogram and Valentini's nomogram

Nomograms developed with clinical information and proposed by Valentini et al., which could be used for DMFS prediction.

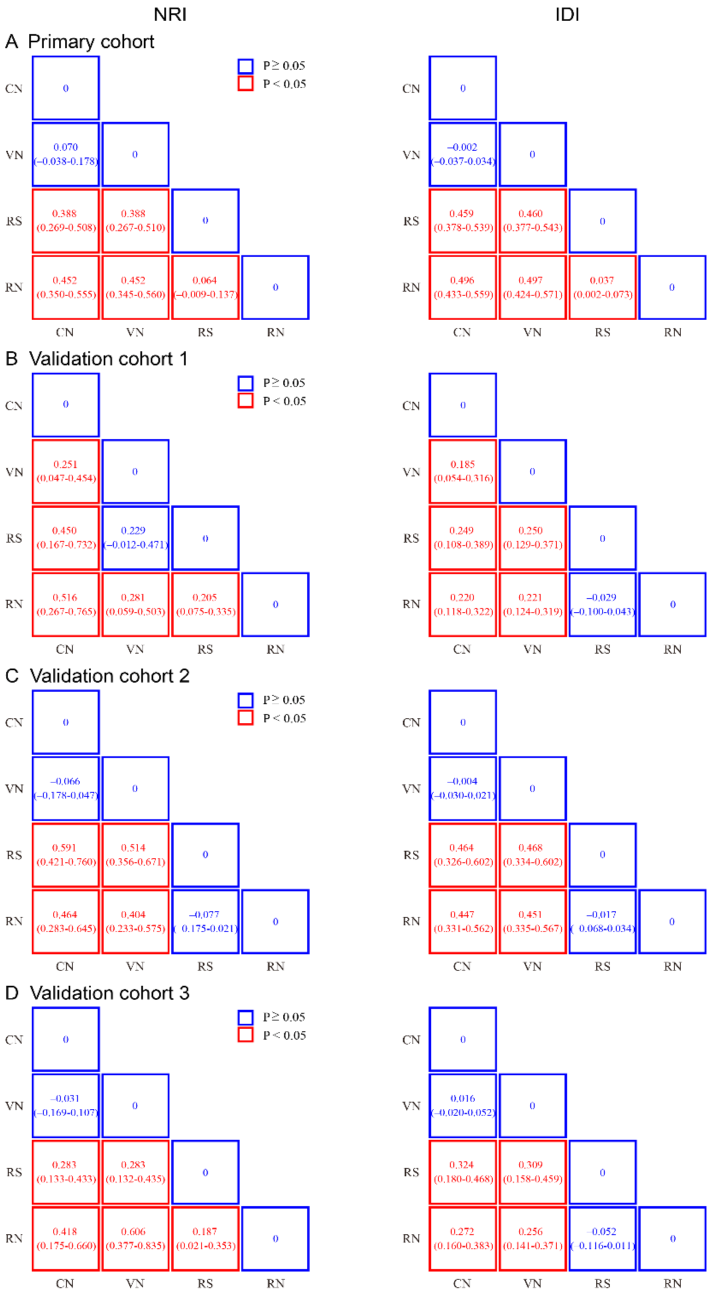

Supplementary Figure 5. Performance improvement evaluation of different models for DMFS with NRI and IDI

The NRI (Left) and IDI (Right) values (95%CI) of different models were shown inside the blocks, the responding significances were shown as the color of the blocks (blue for p≥0.05 and red for p<0.5).

(A) Primary cohort (n = 176), (B) Validation cohort 1 (n = 154), (C) Validation cohort 2 (n = 150), and (D) Validation cohort 3 (n = 149),

CN: clinical nomogram; VN: Valentini's nomogram; RS: radiomic signature; RN: radiomics nomogram; CI: confidence interval.

Supplementary Tables:

Supplementary Table 1. Imaging features selected for construction of the radiomic signature

Features	Sequence	Normalized Values (mean±standard deviation)	
		DM cohort	Non-DM cohort
Coif8_FOS_median	ADC	0.897±0.481	1.458±0.605
Ori_Surface_to_volume_ratio	ADC	1.731±1.236	2.545±0.720
Coif4_GLCM_covariance	ADC	1.531±0.767	2.283±0.619
Coif1_GLCM_autocorrelation	ADC	1.217±0.824	1.914±0.979

Abbreviations: ADC, apparent diffusion coefficient; fos, first order statistics; GLCM, gray-level co-occurrence matrix; Coif is a 3D Coiflet wavelet transform.

Supplementary Table 2. Univariate and multivariable relationships between DM, RS, and clinical information

Variables	Univariate Cox Regression		Multivariable Cox Regression	
	HR (95% CI)	P	HR (95% CI)	P
Radiomic Signature	6.628 (4.434-9.908)	2.96×10^{-20}*	6.109 (4.027-9.268)	1.73×10^{-17}*
Age	0.985 (0.966-1.005)	0.136		
Sex	1.025 (0.607-1.730)	0.926		
clinical stage	1.541 (0.915-2.595)	0.104		
clinical T stage	1.317 (0.849-2.044)	0.219		
Lymph node status	1.469 (0.873-2.471)	0.148		
Location	1.078 (0.758-1.534)	0.676		
Neoadjuvant therapy	1.376 (0.820-2.310)	0.227		
Adjuvant Chemotherapy	2.388 (1.238-4.606)	0.009*		
Adjuvant Radiotherapy	1.507 (0.602-3.772)	0.381		
Surgery procedure	1.113 (0.794-1.560)	0.536		
Surgery approach	0.416 (0.216-0.803)	0.009*	0.489 (0.247-0.967)	0.040*
pN stage	2.146 (1.536-2.999)	7.75×10^{-6}*	1.643 (1.172-2.302)	0.004*
pT stage	1.069 (0.758-1.510)	0.703		
LVI	1.524 (0.888-2.616)	0.126		
PNI	1.836 (0.870-3.875)	0.111		

Abbreviations: pN, pathology nodal stage; pT, pathology tumor stage; LVI, lymphovascular invasion; PNI, perineural invasion.

P-values were calculated using two-sided log-rank test.

Supplementary Table 3. MR scanning parameters for the patients

Hospital	Scanner		Sequence	TR/TE (ms)	FOV (mm)	Matrix	Slice Thickness (mm)	Slice Gap (mm)	Slices	Flip Angle	Acquisition Time (min)
HENAN Provincial people Hospital	GE 3.0T Discover MR750	28	T2W1	4104/85	260×260	320×192	5	1	22	111	1min43s
			DWI	2600/70.6-81	360×360	128×130	5	1	44	90	2min57s
	GE 1.5T OPTIMA MR360	1	T2W1	2451/58	380x380	288x256	6	1	20	90	1min42s
			DWI	3708/76.9	380x380	96x128	6	1	20	90	1min33s
	SIEMENS 3.0T TrioTim	4	T2W1	4000/87	340x340	320x272	4	1	21	140	2min2s
			DWI	4500/76	266x340	192x153	4	1	21	90	2min20s
Yunnan Cancer Hospital	Siemens 1.5T (Avanto)	143	T2WI	3200/100	200×200	288×320	4	0.4	20	90°	2min20s
			DWI	4000/87	260×221	113×138	4	0.4	20	90°	2min22s
Cancer Hospital	GE 3.0T (Signa HDxt)	154	T2WI	5200/101	400×240	320×224	6	1	24	120°	1min29s
			DWI	4000/64.9	400×400	128×128	6	1	24	90°	0min36s
Fudan university Cancer Hospital	GE 3.0T	150	T2WI	4240/120	180×180	288×256	3	0.5	22	90°	4min36s
			DWI	4000/67.2	300×300	128×128	6	2	22	90°	1min12s
The Sixth Affiliated Hospital of Sun Yat-sen University	GE1.5T (Optix360)	149	T2WI	4715/129	280×292	320×224	3	1	20	90°	3min23s
			DWI	4818/93	280×292	192×192	5	1	20	90°	2min

Abbreviations: FOV, field of view; TR, repetition time; TE, echo time; T2WI, T2 weighted imaging; DWI, diffusion weighted imaging; CE, contrast enhancement.

Supplementary Reference:

[1] Babyak MA: What you see may not be what you get: a brief, nontechnical introduction to overfitting in regression-type models. Psychosom Med 66:411–421, 2004.

[2] Chalkidou A, O'Doherty MJ, Marsden PK: False Discovery Rates in PET and CT Studies with Texture Features: A Systematic Review. PLoS One 10: e124165, 2015.

[3] Valentini V, van Stiphout RG, Lammering G, et al: Nomograms for predicting local recurrence, distant metastases, and overall survival for patients with locally advanced rectal cancer based on European randomized clinical trials. J Clin Oncol 29:3163-72, 2011.
