# Supplementary material for: Predicting distant metastasis and chemotherapy benefit in locally advanced rectal cancer
Source: Nat Commun. 2020 Aug 27;11:4308. doi: 10.1038/s41467-020-18162-9 (PMC7452897; doi:10.1038/s41467-020-18162-9)
Supplement: Supplementary file 2 — Reporting Summary [file 41467_2020_18162_MOESM2_ESM.pdf]

nature research

Corresponding author(s): Jie Tian

Last updated by author(s): Jul 14, 2020

Reporting Summary

Nature Research wishes to improve the reproducibility of the work that we publish. This form provides structure for consistency and transparency in reporting. For further information on Nature Research policies, see Authors & Referees and the Editorial Policy Checklist .

Statistics

For all statistical analyses, confirm that the following items are present in the figure legend, table legend, main text, or Methods section.

n/a	Confirmed	
☐	☒	The exact sample size (n) for each experimental group/condition, given as a discrete number and unit of measurement
☐	☒	A statement on whether measurements were taken from distinct samples or whether the same sample was measured repeatedly
☐	☒	The statistical test(s) used AND whether they are one- or two-sided<br>*Only common tests should be described solely by name; describe more complex techniques in the Methods section.*
☐	☒	A description of all covariates tested
☐	☒	A description of any assumptions or corrections, such as tests of normality and adjustment for multiple comparisons
☐	☒	A full description of the statistical parameters including central tendency (e.g. means) or other basic estimates (e.g. regression coefficient) AND variation (e.g. standard deviation) or associated estimates of uncertainty (e.g. confidence intervals)
☐	☒	For null hypothesis testing, the test statistic (e.g. F, t, r) with confidence intervals, effect sizes, degrees of freedom and P value noted<br>*Give P values as exact values whenever suitable.*
☒	☐	For Bayesian analysis, information on the choice of priors and Markov chain Monte Carlo settings
☐	☒	For hierarchical and complex designs, identification of the appropriate level for tests and full reporting of outcomes
☐	☒	Estimates of effect sizes (e.g. Cohen's d, Pearson's r), indicating how they were calculated

Our web collection on statistics for biologists contains articles on many of the points above.

Software and code

Policy information about availability of computer code

Data collection	All patients underwent a conventional rectal MRI protocol that included DWI and T2WI, the scanners including GE 3.0T, GE 1.5T, SIEMENS 3.0T, SIEMENS 1.5T.
Data analysis	The data analysis were performed using R software (version 3.5.2), Matlab (version R2017b), IBM SPSS (version 25.0). Source code of proposed method can be found in the following Github repository, https://github.com/SK94-ai/Radiomics-Predicting-Distant-Metastasi.

For manuscripts utilizing custom algorithms or software that are central to the research but not yet described in published literature, software must be made available to editors/reviewers. We strongly encourage code deposition in a community repository (e.g. GitHub). See the Nature Research guidelines for submitting code & software for further information.

Data

Policy information about availability of data

All manuscripts must include a data availability statement. This statement should provide the following information, where applicable:

- Accession codes, unique identifiers, or web links for publicly available datasets
- A list of figures that have associated raw data
- A description of any restrictions on data availability

The data generated and analyzed during the current study are available from the corresponding author on reasonable request. The source data underlying Figs. 1–2, 4-6, Supplementary Figs. S2–S3, S5, and Table 2 is provided as a Source Data file.

Field-specific reporting

Please select the one below that is the best fit for your research. If you are not sure, read the appropriate sections before making your selection.

Life sciences study design

All studies must disclose on these points even when the disclosure is negative.

Sample size	For the sample size evaluation of the primary cohort, the rule of thumb is that the number of predictors should remain within 1/15–1/10 of the sample size in the training dataset (1, 2). In this study, 4 imaging features were selected for radiomic signature, while the sample size of the primary cohort was 176. Thus, the relative between the number of patients in the primary cohort and the selected features for the radiomic signature was acceptable. For the sample size evaluation of the validation cohort, a sample size estimation method proposed in the book "Sample Size Calculations in Clinical Research 2nd Ed" based on F test was performed.<br>Reference:<br>1. Babyak MA, What you see may not be what you get: a brief, nontechnical introduction to overfitting in regression-type models. Psychosom Med, 2004; 66: 411–421.<br>2. Chalkidou A, O'Doherty MJ, Marsden PK. False Discovery Rates in PET and CT Studies with Texture Features: A Systematic Review. PLoS One, 2015; 10: e124165.
Data exclusions	a) Patients with a history of cancer <5 years;<br>b) Patients with residual tumor and/or circumferential resection margin involvement;<br>c) Patients with <3 years of follow-up data;<br>d) Patients who did not undergo postoperative imaging or clinical follow-up to detect recurrence.
Replication	The reproducibility of the model was verified in the three independent validation cohort.
Randomization	The patients were divided into four cohorts according to different centers: the primary cohort (n = 176 from centers 1 and 2) and three external validation cohorts (validation cohort 1: n = 154 from center 3, validation cohort 2: n = 150 from center 4, and validation cohort 3: n = 149 from center 5). This allocation would not be relevant to our study for the distant metastasis was independent of the centers.
Blinding	The investigators were blinded to group allocation.

Reporting for specific materials, systems and methods

We require information from authors about some types of materials, experimental systems and methods used in many studies. Here, indicate whether each material, system or method listed is relevant to your study. If you are not sure if a list item applies to your research, read the appropriate section before selecting a response.

Materials & experimental systems

n/a	Involved in the study	
☒	☐	Antibodies
☒	☐	Eukaryotic cell lines
☒	☐	Palaeontology
☒	☐	Animals and other organisms
☐	☒	Human research participants
☒	☐	Clinical data

Methods

n/a	Involved in the study	
☒	☐	ChIP-seq
☒	☐	Flow cytometry
☒	☐	MRI-based neuroimaging

Human research participants

Policy information about studies involving human research participants

Population characteristics	The collected characteristics of patients including three types, the detailed characteristics were as following:<br>a) Clinical characteristics, including sex (male/female = 406/223), age at surgery (56.5±11.8), clinical stage (c stage=II, n=205; c stage=III, n=424), clinical tumor (cT) stage (cT=II, n=11; cT=III, n=434; cT=IV, n=184), lymph node (LN) status (LN-negative, n=188; LN-positive, n=441) and tumor location (>10cm, n=157; 5-10cm, n=289; <5cm, n=183).<br>b) Treatment factors, including preoperative therapy protocol (yes, n=384; no, n=245), surgical procedure (Dixon and preventive ileostomy, n=115; Dixon, n=288; Miles, n=196; Hartmann, n=30), surgical approach (laparoscopic resection, n=320; open resection, n=309), and fluorouracil-based adjuvant chemotherapy (yes, n=406; no, n=223), postoperative radiotherapy (yes, n=40; no, n=589).<br>c) Pathological factors, including tumor stage (pT=0, n=66; pT=1/2, n=106; pT=3/4, n=457) and nodal stage (pN=0, n=359; pN=1, n=174; pN=2, n=96). The pathological factors were judged based on the 8th edition of the AJCC Cancer Staging Manual and the NCCN guidelines.
Recruitment	This study is a retrospective study. The patients were recruited according to the following inclusion criteria:<br>a) Primary rectal adenocarcinoma confirmed via biopsy;<br>b) Locally advanced disease (stage II or III) determined tumor based on pre-treatment computed tomography of the chest and abdomen and pelvis magnetic resonance imaging, according to the 8th edition of the AJCC Staging Manual;<br>c) A tumor that was located within 12 cm of the anal verge;<br>d) Age of 18-75 years;<br>e) No other treatment before the MRI scan;<br>f) MRI scan including diffusion-weighted MRI, T2-weighted MRI;<br>g) Radical surgery was performed.
Ethics oversight	The study's protocol was approved by the ethics committee of Yunnan Cancer Hospital, the ethics committee of Henan Provincial People's Hospital, the ethics committee of Cancer Hospital Chinese Academy of Medical Sciences, the ethics committee of Fudan University Shanghai Cancer Center and the ethics committee of Guangdong Gastrointestinal Hospital.

Note that full information on the approval of the study protocol must also be provided in the manuscript.
